# Construction of gene clusters resembling genetic causal mechanisms for common complex disease with an application to young-onset hypertension

**DOI:** 10.1186/1471-2164-14-497

**Published:** 2013-07-23

**Authors:** Ke-Shiuan Lynn, Chen-Hua Lu, Han-Ying Yang, Wen-Lian Hsu, Wen-Harn Pan

**Affiliations:** 1 Institute of Information Science, Academia Sinica, Taipei, Taiwan; 2 Institute of Biomedical Sciences, Academia Sinica, Taipei, Taiwan; 3 National Health Research Institutes, Mialoli, Taiwan

**Keywords:** Genetic causal mechanism, Sufficient cause, Data-mining, Young-onset hypertension, Complex disease

## Abstract

**Background:**

Lack of power and reproducibility are caveats of genetic association studies of common complex diseases. Indeed, the heterogeneity of disease etiology demands that causal models consider the simultaneous involvement of multiple genes. Rothman’s sufficient-cause model, which is well known in epidemiology, provides a framework for such a concept. In the present work, we developed a three-stage algorithm to construct gene clusters resembling Rothman’s causal model for a complex disease, starting from finding influential gene pairs followed by grouping homogeneous pairs.

**Results:**

The algorithm was trained and tested on 2,772 hypertensives and 6,515 normotensives extracted from four large Caucasian and Taiwanese databases. The constructed clusters, each featured by a major gene interacting with many other genes and identified a distinct group of patients, reproduced in both ethnic populations and across three genotyping platforms. We present the 14 largest gene clusters which were capable of identifying 19.3% of hypertensives in all the datasets and 41.8% if one dataset was excluded for lack of phenotype information. Although a few normotensives were also identified by the gene clusters, they usually carried less risky combinatory genotypes (insufficient causes) than the hypertensive counterparts. After establishing a cut-off percentage for risky combinatory genotypes in each gene cluster, the 14 gene clusters achieved a classification accuracy of 82.8% for all datasets and 98.9% if the information-short dataset was excluded. Furthermore, not only 10 of the 14 major genes but also many other contributing genes in the clusters are associated with either hypertension or hypertension-related diseases or functions.

**Conclusions:**

We have shown with the constructed gene clusters that a multi-causal pie-multi-component approach can indeed improve the reproducibility of genetic markers for complex disease. In addition, our novel findings including a major gene in each cluster and sufficient risky genotypes in a cluster for disease onset (which coincides with Rothman’s sufficient cause theory) may not only provide a new research direction for complex diseases but also help to reveal the disease etiology.

## Background

Effective mapping of complex disease genes is one of the major goals of genomic research. With advancements in genomic technology, the genome-wide association study (GWAS) approach has been adopted to identify novel genes for common complex diseases owing to its ability to simultaneously examine a large number of polymorphism-phenotype associations [1-4]. Although GWAS have indeed identified certain susceptibility genes for many diseases, the genes thus far discovered mostly have been associated with small to modest effects [1-5]. For very complex diseases, such as hypertension, GWAS have revealed very few genes despite a large number of patients that have been studied. It is generally accepted that common complex disease etiologies are heterogeneous in nature [5-8]. In “state of art” GWAS approach, however, inheritance models involving gene-gene interactions and gene-environment interactions [9-13] have not been taken into consideration.

Rothman’s concept of sufficient causes [14,15] describes scenarios in which multiple causal mechanisms can all lead to the development of a disease. Each mechanism is depicted as a causal pie, composed of several component causes, and the number of causes varies in these mechanisms. These component causes—genetic or environmental—can be shared or completely different across causal mechanisms. Thus, the probability of developing a disease is increased as a person carries more and more component causes. Under such a conceptual framework, if a gene is only involved in few of the causal pies which explain only a fraction of disease population, its effect toward a disease could be insignificant when all patients are considered. This model provides an explanation for low reproducibility across studies. Although Rothman’s causal pie model is well known in epidemiology, few attempts have been made to construct such pies, not to mention its recognition and application in the genetic field.

In the present study, we focused on constructing gene clusters resembling genetic causal pies using genome-wide single-nucleotide polymorphism (SNP) data for young-onset hypertension (YOH), which has a stronger genetic attribute than its late-onset counterpart [16,17]. We made use of two large Caucasian databases, the Framingham Heart Study (FHS, http://www.framinghamheartstudy.org[18] and Wellcome Trust Case Control Consortium (WTCCC, http://www.wtccc.org.uk[19]), and two large Taiwanese databases, the Taiwan Young-Onset Hypertension Study (Taiwan YOH [17]) and Taiwan Han Chinese Cell and Genome Bank (THCCG, http://ncc.sinica.edu.tw/han-chinese_genomebank[20]). We aimed to find either single SNPs or multiple SNP sets each of which resembles a genetic causal pie and could distinguish a certain proportion of hypertensives (HTs) from normotensive controls (NCs). Owing to limited databases and many gene clusters found in the databases, we intended to demonstrate the existence of such causal pie-like gene clusters rather than to construct all the genetic causal pies. We thus developed an algorithm to construct influential (as many as patients being identified) and effective (cluster components identifying the same group of patients) gene clusters. We first searched for pair-wise gene-gene interactions primarily observable in FHS and Taiwan YOH patients via an exhaustive search. Gene (SNP) pairs that identified similar patients were further merged into clusters following the logic of the multiple genetic causal pies framework. The resulting gene clusters were then tested for reproducibility on various platforms (including gene expression data) and examined for robustness in varied algorithm parameters. Crucial gene pairs that represented minimum and sufficient component causes in each of the genetic causal pies were searched, and their effects to hypertension onset were discussed. Moreover, influential functions, process and pathways of these genes were collated to shed light on hypertension etiology.

## Methods

This study was approved by the Internal Review Board of Academia Sinica. All four databases used in this paper were approved by local institutional review boards or equivalent committees and all participants in the databases signed a written informed consent at all institutions/hospitals where they were recruited and human experimentation was conducted.

### Characteristics of the four employed databases n

The FHS database contains 7,126 subjects (Framingham, Massachusetts, U.S.A., predominantly Caucasian) among whom 6,748 were assayed by the Affymetrix500k platform with detailed information on blood pressure measurements and medications. The WTCCC database currently consists of datasets from three studies (WTCCC1~WTCCC3). However, only the dataset from WTCCC1 was available at the time our experiment was conducted. The dataset includes 2,001 hypertensive cases and 3,004 NCs (1504 from the 1958 British Birth Cohort and 1500 from the UK Blood Service Control Group), all from the British population and assayed by the Affymetrix500k platform. The Taiwan YOH database contains 1,023 well-characterized YOH subjects, among which 175 were assayed by the Affymetrix100k platform, 200 were assayed by the Affymetrix500k platform, and 400 were assayed by the Illumina550k platform. The THCCG database involved 3,435 sampled residents with detailed clinical information. Among them, 175 were assayed by the Affymetrix100k platform, 468 were assayed by the Affymetrix500k platform, and 1,000 were assayed by the Illumina550k platform.

### Training and test datasets

We extracted suitable samples from the four databases to construct our training and test datasets. To prevent ambiguous data from disrupting our data mining–based approach, NC subjects in FHS and THCCG subjects with multiple high blood pressure readings (≥120/80 mmHg) were removed from the datasets. To ensure a strong genetic effect on the onset of hypertension, late-onset (onset >50 years) and secondary HT patients were also excluded. Detailed inclusion criteria for HT patients and for NC subjects are listed in Additional file 1: Method S1. In addition, we adopted the “SNP Finder” in SNPper (http://snpper.chip.org/bio/snpper-enter[21]) to search for intragenic SNPs and their corresponding gene symbols in each genotyping platform. The resultant training and test datasets are summarized below and detailed subject IDs are provided in Additional file 2.

Training datasets:

(1) Caucasian subset (FHS_Affy500k): Affymetrix500k genotype data extracted from FHS, including 214,383 intragenic SNPs for 3,186 Framingham residents, among whom 305 developed hypertension and 2881 remained normotensive during follow-up

(2) Taiwanese subset (Taiwan_Affy500k): Affymetrix500k genotype data, including 213,353 intragenic SNPs for 200 HT cases from the Taiwan YOH study and 184 NC subjects from THCCG

Test datasets:

(1) Caucasian subset (WTCCC_Affy500k): Affymetrix500k genotype data extracted from WTCCC, including 214,383 intragenic SNPs for 2,001 HT cases and 3,004 NC subjects

(2) Taiwanese subsets:

(a) Taiwan_Illu550k: Illumina550k genotype data, including 221,828 intragenic SNPs for 200 HT cases from the Taiwan YOH study and 400 NC subjects from THCCG

(b) Taiwan_Affy100k: Affymetrix100k genotype data, including 47,038 intragenic SNPs for 129 HT cases from the Taiwan YOH study and 129 NC subjects from THCCG

Some of the subjects overlapped in the Taiwan_Illu550k and Tai-wan_Affy100k, leaving a total of 266 unique HT cases and 446 unique NC subjects in the Taiwan test dataset (see Additional file 1: Figure S1 for detailed calculations). To demonstrate reproducibility among genotyping platforms, these overlapped subjects were not removed from the two test datasets because the adopted SNPs differed be-tween the two platforms, and some patients may have been identified by one of them. However, the overlapped subjects were counted only once for the evaluation of classification performance.

More importantly, because we do not have phenotype information for WTCCC, late-onset (WTCCC recruited HT patients < 60 yr of age but we required ≤ 50 yr of age) may have been included in the HT subset, whereas high body mass index, high blood sugar, or border-line blood pressure (120/80~140/90 mmHg) subjects may have been included in the NC subset. For comparison, in FHS_Affy500k, only 305 of 557 (54.8%) HT patients and 2,881 of the remaining 6191 (46.5%) subjects who had genotype data and satisfied our inclusion criteria were selected from the FHS database. Therefore, although the WTCCC_Affy500k was used as one of the test datasets, focus should be placed on the reproducibility of the constructed gene sets in its HT population instead of on its classification accuracy.

### Detection of gene-gene interaction

Several definitions of gene-gene interaction (or epistasis) have been proposed in the literature [11-13,22]. Based on these definitions, many methods have also been developed to detect gene-gene interactions. These methods can be roughly categorized into three classes: exhaustive search, regression-based approach, and data-mining approach. Exhaustive search, which performs a certain test for all possible pairs in the dataset, is the simplest way to detect interactions [23]. However, such a method is not suitable for higher-order interactions since the number of tests grows exponentially and soon becomes computationally infeasible. Popular in statistical analysis packages, regression-based approaches attempt to fit a regression model (linear, logistic, or logic) between subjects’ multilocus genotypes and their outcomes and to test whether the effect of multiplicative terms is negligible [24-26]. In contrast to the previous two approaches, data-mining approaches are preferred for detecting high dimensional interactions. They focus on selecting a minimal subset of loci so that, in the subspace spanned by the loci, a hyperplane or a hypersurface can be constructed to distinguish different outcome groups. Examples of this category are multifactor-dimensionality reduction (MDR) [27], combinatorial partitioning method (CPM) [28], genetic programming [29], neural networks [30] and ort vector machines [31]. Other methods, including Bayesian model-based approach [32] and entropy-based approach [33], have also been developed.

**Figure 1:**
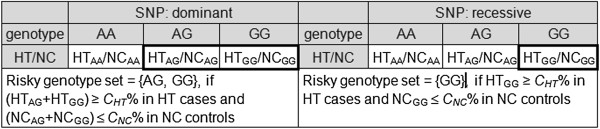
**Criteria for a risky genotype set of a single disease gen (SNP).** In the above example, the SNP is AG polymorphism with disease allele G.

In our preliminary studies, we observed that many interacting genes have shared genes. Also, gene pairs with a shared gene often identified a similar group of individuals and thus can be organized together to form a gene cluster anchored by a major gene. To detect all such clusters and their component genes in a genome-wide data, a method that can quickly detect all the possible interacting gene pairs is needed. To this end, we adopted an exhaustive search with simple testing criteria to detect single genes and interacting gene pairs that are associated with increased risk. We first define the following terms that were used in our detection method:

**Risky genotype set**: certain genotypes (as illustrated in Figure 1, each as a risky genotype) that are observable in at least *C*_*HT*_% (*C*_*HT*_ > 0) of a diseased population and at most *C*_*NC*_% (*C*_*HT*_ >*C*_*NC*_ ≥ 0) of a non-diseased population

**Single disease gene**: a single gene that exhibits a risky genotype set

**Figure 2:**
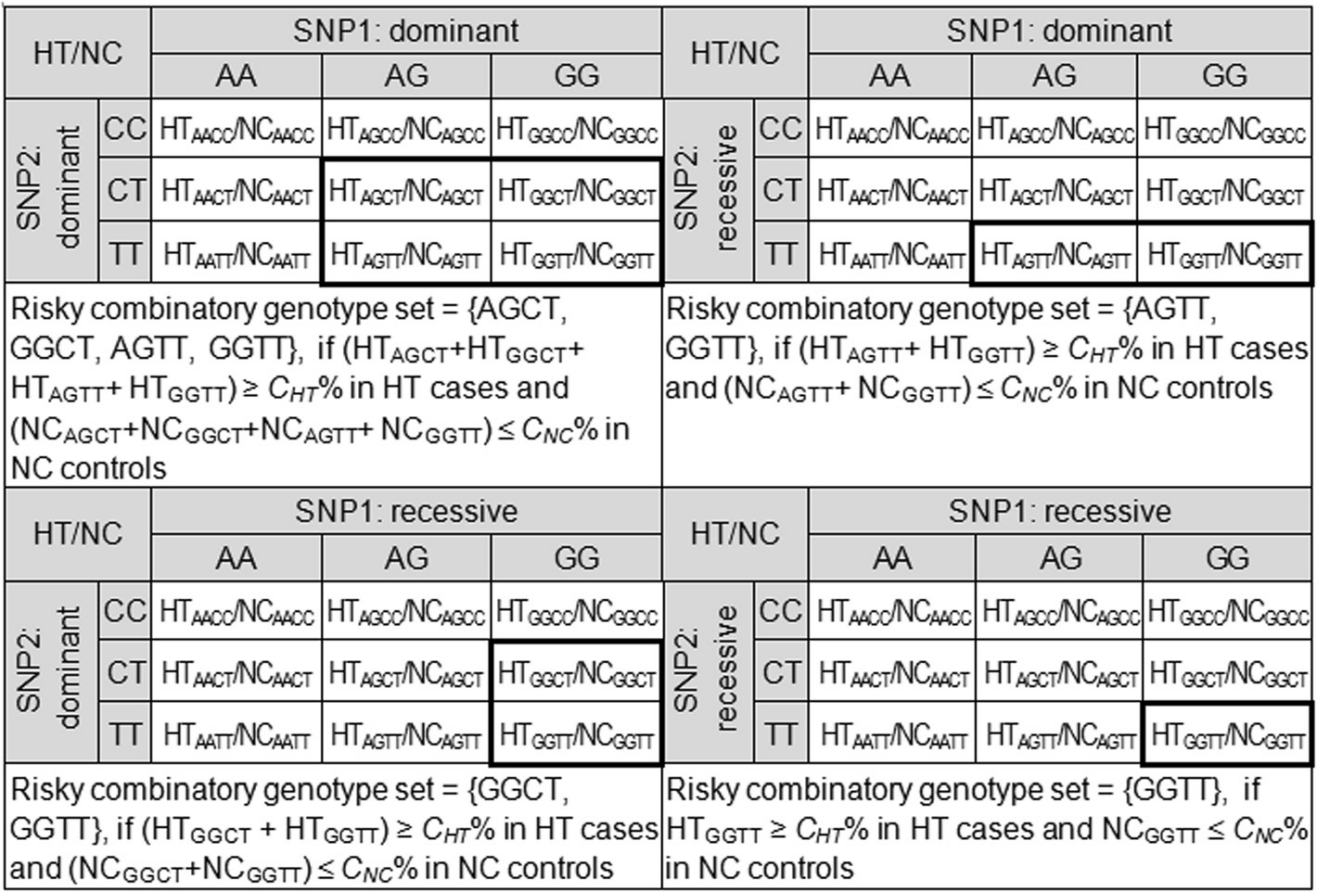
**Criteria for a risky combinatory genotype set of a disease gene (SNP) pair.** In the above example, SNP1 is the AG polymorphism with disease allele G whereas SNP2 is the CT polymorphism with disease allele T.

**Risky combinatory genotype set**: certain combinatory genotypes (as illustrated in Figure 2, each as a risky combinatory genotype) that are observable in at least *C*_*HT*_% (*C*_*HT*_ > 0) of a diseased population and at most *C*_*NC*_% (*C*_*HT*_ >*C*_*NC*_ ≥ 0) of a non-diseased population

**Gene-gene interaction**: a pair of genes that exhibit a risky combinatory genotype set without either of them being a disease gene

**Disease gene pair**: a pair of genes that exhibit a gene-gene interaction

To identify disease genes and disease gene pairs, we exhaustively searched for all the risky genotype sets for all SNPs and then search for all the risky combinatory genotype sets for all SNP pairs. We noted that, the value of *C*_*NC*_ was set to a small value instead of zero in real applications to tolerate possible genotyping and sampling errors in the dataset. In addition, we adopted the ceiling function, ⌈*x*⌉ = min{*m* ∈ *Z*| ≥ *x*}, in our algorithm to deal with the fraction resulting from the product of the criterion and sample size. Such a design allowed more qualified gene and gene pairs for datasets with a small NC population where genotyping and sampling qualities usually exhibit large variations. Also, although we used SNP data to construct genetic clusters, we will merge them by the associated genes for the subsequent cross-platform comparisons and function analysis.

### The gene cluster construction algorithm

Two problems were encountered as we attempted to organize the detected gene pairs into gene clusters: (i) value assignment for *C*_*HT*_ and *C*_*NC*_, and (ii) removal of false positive gene pairs. For stringent detection criteria, i.e. a very large *C*_*HT*_ with a very small *C*_*NC*_, the detected disease genes and gene pairs can be too conservative to provide clear information about the underlying disease mechanism. However, as the criteria were relaxed, the detected disease genes and gene pairs increased quickly and soon became unmanageable. To solve this dilemma, we proposed first using stringent criteria to generate a manageable amount of candidates, and then relaxing the criteria to search for additional gene pairs for each gene cluster. On the other hand, false positive gene pairs in a gene cluster degenerate its classification performance and provide false information to the underlying disease mechanism. Although two gene pairs with a shared gene may not identified identical individuals, those identified by a gene cluster usually carry more risky combinatory genotypes in the gene cluster than the others (see Additional file 1: Figure S2 for a demonstration). Therefore we proposed accumulating multiple gene pairs that have a shared gene so as to locate the frequently identified subjects (FIS) and then to remove false positive gene pairs that identified subjects other than these FIS. The gene clusters formed by our algorithm is rather intrinsic in the datasets and may resemble Rothman’s genetic causal pies. Furthermore, we have proven in Additional file 1: Method S2 that the probability of a false positive gene cluster containing *k* non-linkage disequilibrium (non-LD) SNP pairs and identifying *m* subjects in a population of *n* subjects is bounded above by (*m*/*n*)^*k*^.

Our algorithm consists of three stages: cluster selection, component growth and component pruning. During the first stage, we set up a set of stringent criteria to identify influential disease gene pairs and grouped them with shared genes. Then at the second stage, we iteratively relaxed the criteria to encourage effective gene clusters to include additional gene pairs until new gene pairs started to identify different groups of HT patients. Finally, at the third stage, all disease gene pairs that identified different groups of patients were removed from the cluster. In this work, we used *C*_*HT*_ = 2.0 and *C*_*NC*_ = 0.1 to produce manageable cluster size in the first stage. Let the sample size of the HT and NC populations be *S*_*HT*_ and *S*_*NC*_, respectively. The proposed gene cluster construction algorithm comprises the following steps.

Step 1 Cluster selection. Select a conservative set of gene clusters using stringent criteria:

Step 1.1 Set *t*_*HT*_ = *t*_*HT*0_ = ⌈*S*_*HT*_ × *C*_*HT*_*%*⌉ and *t*_*NC*_ = *t*_*NC0*_ = ⌈*S*_*NC*_ × *C*_*NC*_*%*⌉ and use them to replace *C*_*HT*_ and *C*_*NC*_ in Figures 1 and 2.

Step 1.2 Search for all single genes with risky genotype sets (as illustrated in Figure 1) from the training datasets.

Step 1.3 Search for all gene pairs with risky combinatory genotype sets (as illustrated in Figure 2) from the training datasets.

Step 1.4 Find shared genes among the qualified gene pairs and use them to group the gene pairs.

Step 2 Component growth: For each constructed gene cluster, repeat the following steps until the HT patients identified by the new disease gene pairs differ from those by the existing ones:

Step 2.1 Set *t*_*HT*_ = *t*_*HT*_ - 1 and *t*_*NC*_ = *t*_*NC*_ + 1.

Step 2.2 Search for additional gene pairs with risky combinatory genotype sets from the training datasets.

Step 2.3 For each constructed gene cluster, record subject IDs that are frequently identified by its gene pair components:

Step 2.3.1 Locate the most frequently identified subjects (MFIS).

Step 2.3.2 Select subjects that were identified at least 1/4 the time compared with the MFIS, and categorize them as frequently identified subjects (FIS).

Step 2.3.3 Select the *t*_*HT*_ most frequently identified subjects if the number of FIS is less than *t*_*HT*_.

Step 3 Component pruning:

Step 3.1 Select gene clusters with sufficient number (≥ *t*_*HT*0_) of FIS.

Step 3.2 For each gene cluster, remove the gene pairs which identify subjects not in FIS.

### Identification of influential genes using gene expression data

The gene clusters constructed from the SNP data usually consist of many genes which is disadvantageous for etiology analysis. We attempted to identify the influential genes using expression data. We selected 253 (135 in Taiwan_Affy500k and 118 in Taiwan_Illu550k) HT patients and 232 (36 in Taiwan_Affy500k and 196 in Tai-wan_Illu550k) NC subjects who had gene expression data for the demonstration. For each subject, three replicates of genome-wide expression data were generated by the following three steps: (i) lymphocytes were isolated from the fasting blood immediately after it was drawn; (ii) the lymphoblastoid cell line was established via Epstein-Barr virus transformation; (iii) total RNA was extracted and hybridized onto three Phalanx Human OneArrays (HOA v5.1, Phalanx Biotech Group, Taiwan), each of which contains 39,200 polynucleotide probes with 25,215 of them mapped to the latest draft of the human genome.

Before merging the three replicates for each subject, we checked the consistency among them. We first computed the Pearson correlation coefficient for every two replicates and removed those with at least one correlation less than 0.9. We then checked the consistency for each gene if more than one of the replicates were available. The values of a gene were set to 0 (missing) if its minimum was less than 60% of its maximum. After such an adjustment, replicates were merged using median values. A base-2 logarithm and Z-score global normalization were applied to the merged data. In the resultant data, we further set those values higher than 6 to 0 (missing) since they were outliers or represented false signals.

We developed an algorithm to identify influential genes in each gene cluster using the above gene expression data. Starting with the shared gene in a gene cluster, the algorithm iteratively added a gene in the gene cluster such that the HT patients carrying risky combinatory genotypes can be maximally discriminated (in terms of adjusted *p* values) from HT patients without carrying risky combinatory genotype, from NC subjects carrying risky combinatory genotype and from NC subjects without carrying risky combinatory genotype. This process is stopped if no better discrimination can be achieved by adding any other gene in the gene clusters. Pseudo code of this algorithm is provided in Additional file 1: Method S3. Although such a sequential search may not obtain the best discrimination among the above subject groups, it was adopted for its capability of selecting a small set of influential genes so that the underlying disease mechanisms in a gene cluster can be easily revealed.

## Results

### The constructed gene clusters

No single gene was found to fulfill the stringent criteria, i.e., carrying risky genotype sets in at least 2.0% of HT patients and at most 0.1% of NC subjects in the training datasets. However, allele “CC” of rs16854417, an intronic SNP in SLC9A9, identified 3/305 (0.98%) and 4/200 (2%) of HT patients in FHS_Affy500k and in Taiwan_Affy550k, respectively (see Additional file 1: Table S1 for its allele frequencies in various datasets). In contrast, no NC subject in FHS and only one NC subject in Taiwan_Affy550k carried the CC allele for this SNP. Although this subject was a 53-year-old female with three normal blood pressure readings (120/78, 118/76 and 118/78 mmHg), she had a family history of hypertension.

In search for disease gene pairs, at the cluster-selection stage, we applied the stringent criteria and obtained 264 gene pairs in 360 genes, of which 24 were shared by multiple gene pairs. The 264 gene pairs were then grouped into 103 gene clusters, of which 24 consisted of multiple gene pairs and the remaining 79 contained only one gene pair. At the component growth stage, the criteria were relaxed accordingly for each of the 103 gene clusters to search for additional gene pairs. For the 79 two-gene clusters, the expansion was carried out twice, each assuming that one of the two genes was a shared gene. As a result, the original 103 gene clusters were expanded to 182 gene clusters. Because small gene clusters were more likely to be false positives (see Additional file 1: Method S2 for the proof), we selected the 14 largest gene clusters which contained 17,170 gene pairs in 8,559 genes for the subsequent analysis. The 14 gene clusters were finally reduced to 17,115 gene pairs in 8,524 genes at the component pruning stage. We list in Additional file 1: Table S2 the numbers of overlapping genes between gene clusters as a distance measure. The average percentage of overlapping genes in the 14 gene clusters is 4.9%. Such a low overlapping ratio is expected because the patients identified by the gene clusters exhibited few overlaps. We also provide in Additional files 2 and 3 the gene symbols and SNP rs numbers in the gene clusters obtained at the cluster select stage and at the component pruning stage, respectively.

**Figure 3:**
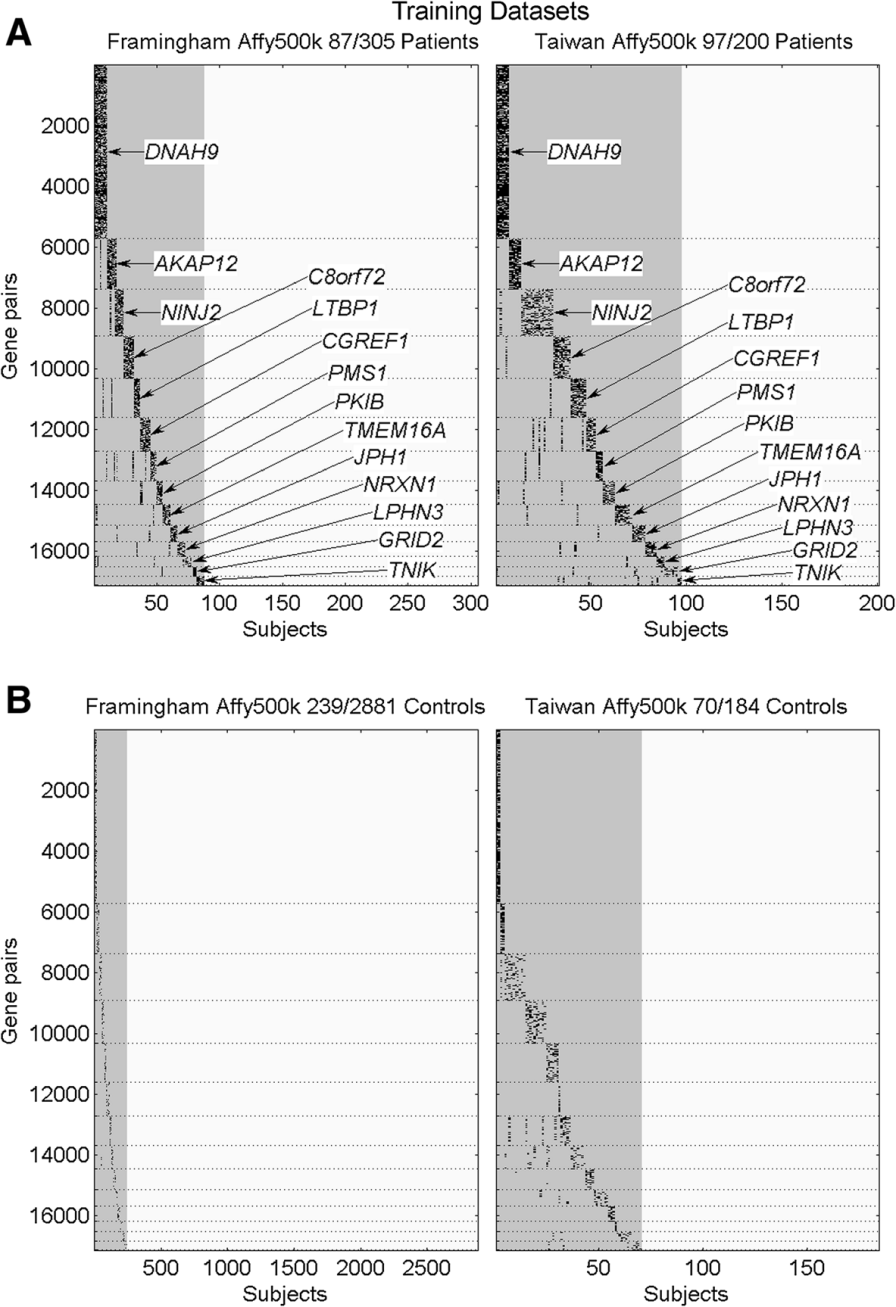
**The 14 gene-subject clusters (denoted by their shared genes) for (A) HT patients and for (B) NC subjects in the two training datasets, FHS_Affy500k (left) and Taiwan_Affy500k (right).** The numerator in the title indicates the number of subjects identified by all gene clusters, whereas the denominator denotes the total num-ber of subjects in the dataset. The gray areas indicate the total portion of identified subjects.

**Figure 4:**
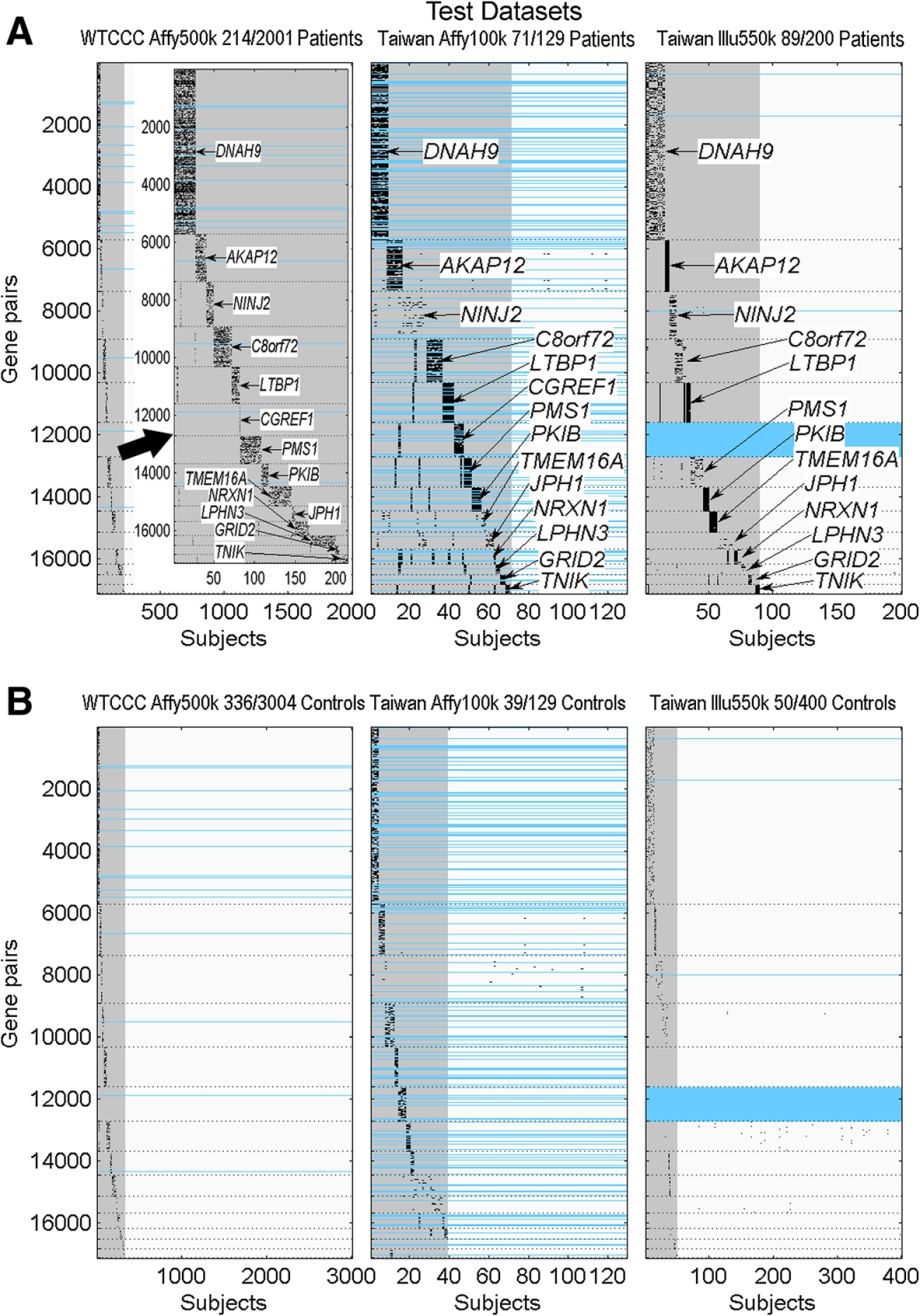
**The 14 gene-subject clusters (denoted by their shared genes) for (A) HT patients and for (B) NC subjects in the three test datasets, WTCCC_Affy500k (left), Taiwan_Affy100k (middle) and Tai-wan_Illu550k (right).** The numerator in the title indicates the number of subjects identified by all gene clusters, whereas the denominator denotes the total number of subjects in the dataset. The gray areas indicate the total portion of identified subjects, whereas the light-blue horizontal lines denote that no corresponding gene pairs could be found in the dataset.

In Figures 3 and 4, we demonstrate how the constructed gene clusters (denoted by their shared genes) identified different groups of subjects in training datasets and in test datasets, respectively, using a cluster visualization procedure (Additional file 1: Method S4). In both figures, the horizontal axis denotes the number of subjects, and the vertical axis denotes the number of gene pairs. A black pixel in the figures represents a subject who carried a risky combinatory genotype in the corresponding gene pair. Moreover, the gray areas in the figures indicate the portion of subjects carrying risky combinatory genotypes in the 14 gene clusters, whereas the light-blue horizontal lines denote that no corresponding gene pairs could be found in the dataset (due to differences among platforms).

Summarizing from Figures 3 and 4, the percentages of the HT population carrying risky combinatory genotypes in the 14 gene clusters were 36.4% (184/505 of which 87/305 in Caucasian and 97/200 in Taiwanese) in training datasets and 15.5% (352/2,267 of which 214/2,001 in Caucasian and 138/266 in Taiwanese) in test datasets. The lower percentage in the test Caucasian may be due to the inclusion of late-onset patients in the WTCCC_Affy500k dataset. On the other hand, the percentages of NC population carrying the risky combinatory genotypes were 10.1% (309/3,065 of which 239/2,881 in Caucasian and 70/184 in Taiwanese) in the training datasets and 12.3% (425/3,450 of which 336/3,004 in Caucasian and 89/446 in Taiwanese) in test datasets. Detailed percentages for each of the 14 gene clusters in the five datasets are presented in Additional file 1: Table S3.

We used PLINK [34] to compute the p-values of the 14 major genes in order to further demonstrate that the identification capability is from gene-gene interactions rather than from the major genes. Some SNPs flanking these major genes did have very small p-values (even after the conservative Bonferroni adjustment), implying that certain genotypes in these SNPs may have important roles in the disease mechanism and thus they are highly associated with hypertension. However, none of the genotypes is capable of identifying HT patients (refer to Additional file 1: Table S4), suggesting that they still need to work with other genes to facilitate the mechanism. For example, the smallest p-value 4.97×10^-24^ (unadjusted) was obtained from the SNP rs2607943 of NINJ2, the major gene of the 3rd cluster. However, its genotype distribution among the 505 patients (346 AAs, 123 AGs, 26 GGs, and 10 missing) and that among the 3065 controls (2596 AAs, 412 AGs, 28 GGs, and 29 missing) showed that none of the genotypes can be used to identify HT patients. Similarly, although some of the remaining 13 major genes had small p-values (ranging 10^-2^~10^-17^), none of their genotypes was capable of distinguish HT patients from NC subjects.

### Gene clusters resembling genetic causal pies

**Figure 5:**
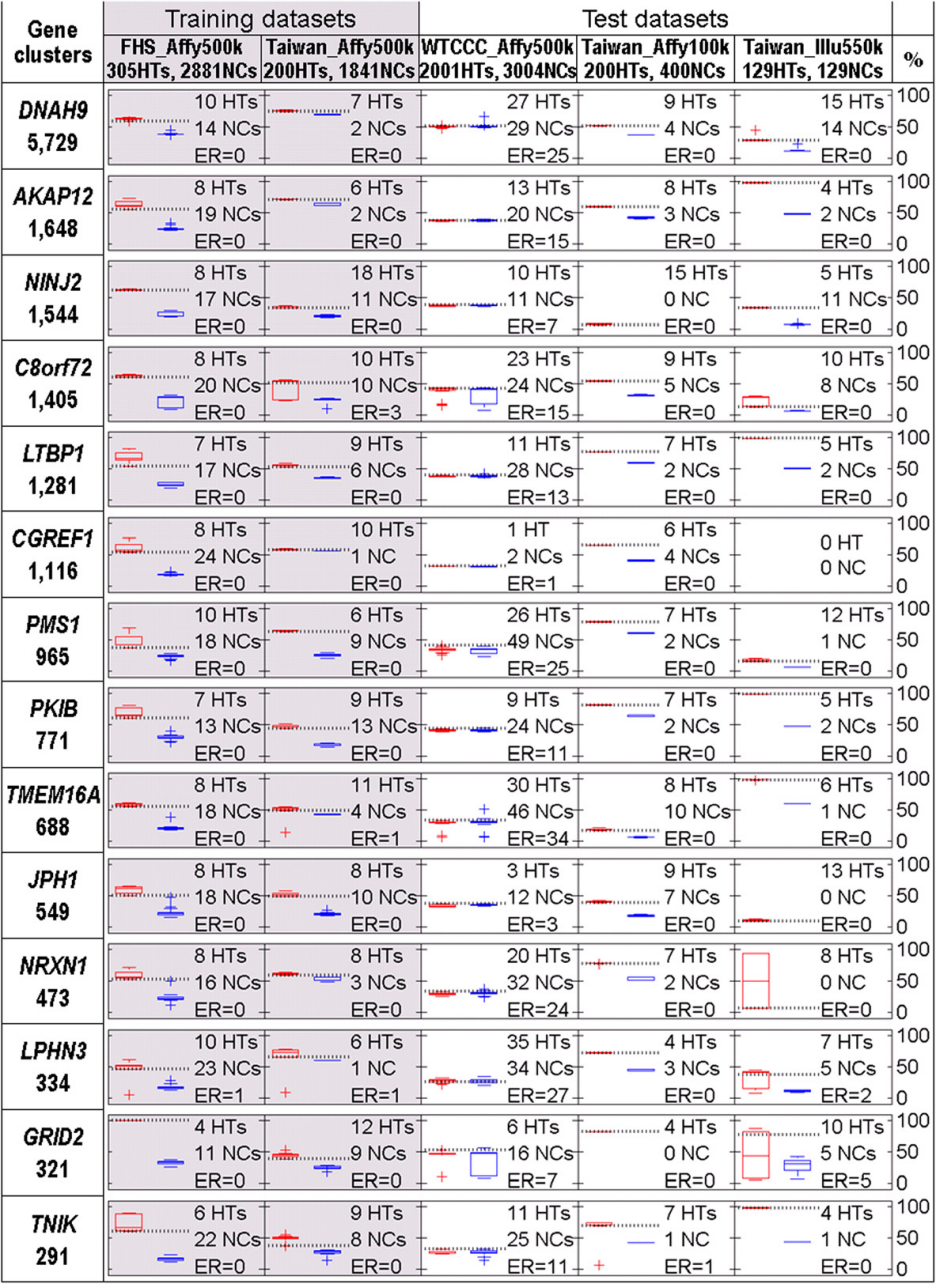
**Box plots of percentage of risky combinatory genotypes carried in the subjects who were identified by one of the fourteen gene clusters in the five datasets.** Each box in the left-hand side shows the shared gene and the number of involved genes in a gene clusters. Red box plots represent HT patients, whereas blue box plots are for NC subjects. The horizontal dashed line represents the cut-off percentage of risky combinatory genotypes for defining HT computed for each data set. ER denotes classification error.

We have shown in Figures 3 and 4 that the 14 constructed gene clusters were capable of identifying higher percentage of HT population than that of NC population and each gene cluster seemed to identify a distinct group of subjects. Further computing the number of risky combinatory genotypes carried in each subject, we found that HT patients usually carried more risky combinatory genotypes than NC subjects. This can be seen in Figures 3 and 4 that the gene-subject clusters for HT patients (part (A)) usually exhibit darker blocks than those for NC subjects (part (B)). In Figure 5, we used box plots to show the distributions of carried risky combinatory genotypes for HT patients (red) and for NC subjects (blue) that are identified by the same gene cluster in a dataset. Due to gene diversity among platforms, we used percentage (with respect to the size of the corresponding gene cluster), rather than number, of carried risky combinatory genotypes to demonstrate the difference between HT patients and HC subjects in the figure. Moreover, for each gene cluster in a dataset, we selected a percentage from the HT patients which resulted in minimum classification error as a threshold for disease onset. In Figure 5, the threshold is represented by a dashed line between HT patients and NC subjects in a box plot, whereas the classification error is denoted by ER.

In addition to platform differences, our datasets also exhibited ethnic differences, i.e. Caucasian and Taiwanese. Although FHS_Affy500k, Taiwan_Affy500k, and WTCCC_Affy500k were all assayed on the Affymetrix500k platform, they represented different ethnic groups and therefore may have different thresholds for disease onset. We adopted two scenarios, S1 and S2, to compute the thresholds in the three datasets: the former assuming different thresholds for different ethnic groups, whereas the latter anticipating one threshold for a platform. With S1, we separated HT patients from NC subjects with 82.8% classification accuracy (sensitivity = 0.68, specificity = 0.93) or with 98.9% accuracy (sensitivity = 0.98, specificity = 1.0) if WTCCC_Affy500k was excluded. In accuracy calculations, a true positive was a HT patient with sufficient risky combinatory genotypes in at least one gene cluster, whereas a true negative indicated a NC subject with insufficient risky combinatory genotypes in all gene clusters. Detailed classification results are provided in Table 1. From the above results, we found that the sufficient risky combinatory genotype was similar to the sufficient cause in Rothman’s causal pie model in that a subject must carry sufficient risky combinatory genotypes (component causes) in a gene cluster (a causal pie) for the onset of HT. In addition, most of the gene clusters were not only consistently observed in all the datasets but also clustered HT patients into distinct groups, suggesting multiple causal mechanisms for HT.

**Table 1:** Classification accuracy after establishing a cut-off percentage of risky combinatory genotypes in each gene cluster

**Datasets**	**Population**	**Classification accuracy S1(S2)**	**Sensitivity S1(S2)**	**Specificity S1(S2)**	**Subjects with risky genotypes**	
					**HT**	**NC**
FHS_Affy500k (training)	Caucasian	99.7%^+^(99.1%)	0.99(0.98)	1.0(0.996)	87	239
Taiwan_Affy500k (training)	Taiwanese	98.2%^+^(93.4%)	0.97(0.97)	1.0(0.89)	97	70
WTCCC_Affy500k (test)	Caucasian	61.6%(62.9%)	0.25(0.26)	0.85(0.87)	214	336
Taiwan_Affy100k & Taiwan_Illu550k (test)	Taiwanese	98.2%(98.2%)	0.97(0.94)	1.0(1.0)	138^*^	89*
**Overall**	**Both**	**82.8%(82.5%)**	**0.68(0.69)**	**0.93(0.93)**	**536**	**734**
**Overall except WTCCC_Affy500k**	**Both**	**98.9%(97.5%)**	**0.98(0.97)**	**1.0(0.98)**	**322**	**398**

S1: threshold is computed for each dataset; S2: threshold is computed for each platform; ^+^Classification accuracy of the dataset evaluated using a five-fold validation procedure exhibit similar result and is presented in Additional file 1: Table S5; *see Additional file 1: Figure S1 for detailed calculations.

### Advantages in comparison with existed gene-gene interaction algorithms

Most of the algorithms for detecting gene-gene interaction dealt with complex model fitting and therefore their application to GWAS data can be very time consuming or simply infeasible. Also, methods that detect gene-gene interactions among multiple loci cannot guarantee global optimal solutions since only limited combinations are explored. In comparison with conventional methods, our algorithm has the following advantages:

#### Our detection algorithm is fast

We used simple testing criteria to detect gene-gene interactions which allowed us to quickly perform exhaustive search for all gene pairs. Using 19779 SNPs in 505 cases and 3065 controls (cluster1 in our training dataset) as an example, our algorithm took 5 hours and 51 minutes to finish all pairwise tests whereas the PLINK with the “fast-epistasis function” spent 8 hours and 43 minutes (more than 10 days for the “epistasis” function).

#### Our gene clustering algorithm is robust

We tested the robustness of our algorithm to criteria, sample size, and prevalence changes, respectively. Detailed testing procedures were described in Additional file 1: Method S5. We showed in Additional file 1: Figure S3 that our algorithm was capable of detecting the same gene clusters either the criterion *C*_*HT*_ was increased from 2.0 to 2.5 (25% increased) or was decreased from 2.0 to 1.8 (10% decreased) or even to 1.5 (25% decreased). However when the criterion was increased to 2.5, it became too stringent and many gene clusters were no longer recoverable from the component growth stage. Thus, our gene cluster construction algorithm was very robust to criteria changes. We also showed in Additional file 1: Figure S4 that the top 15 gene clusters remained >85% unchanged if 70% of the sample size was used (i.e., 30% of the data was randomly removed). The similarity remained around 76% when 50% of the samples were used and was reduced to about 60% as only 20% samples were used. These results showed that our gene cluster construction algorithm was robust to changes in sample size in comparison with many single-SNP analyses [35]. Similar approach was used to test the robustness of our method to changes in disease prevalence. We created a database with varied hypertension prevalence by reducing the HT samples while keeping the NC samples unchanged as in the previous test. In Additional file 1: Figure S5, the top 15 gene clusters remained 81% unchanged if the disease prevalence was decreased to 70% and the cluster similarity was reduced to about 57% as the prevalence was dropped to 20%. Such a result demonstrated that our algorithm was also robust enough to sustain changes in disease prevalence. The robustness of our algorithm is resulted from the design of detecting influential gene clusters (those identify more HTs) at the first stage and is further improved via encouraging consistent gene clusters (those with gene pairs identifying similar HTs) to grow in the latter two stages.

#### Our detection algorithm can deal with risky factor or protective factor or both

Unlike regression models that detect interactions that are associated with both risk factor and protective factor, our method can be assigned to detect interactions that are associated with either risky factor (as demonstrated in this manuscript) or protective factor (i.e., by setting *C*_*NC*_ >*C*_*HT*_ ≥ 0).

#### Our gene clustering algorithm constructs reproducible clusters

Our test results also showed that the constructed gene clusters were reproduced in both ethnic populations and across three genotyping platforms. We have proven that the probability of a false positive gene cluster detected by our algorithm is very low. For a gene cluster containing *k* non-LD SNP pairs and identifying *m* subjects in a population of *n* subjects, such a probability is bounded above by (*m*/*n*)^*k*^.

#### Our gene clustering algorithm can detect patient subgroups

Our observations showed that many interacting pairs identified similar group of patients. Accumulation of these gene pairs allowed us to identify patient subgroups whereas the identified patients help us to eliminate false positive gene pairs. Most of all, we found the gene clusters resemble different genetic causal pies in that subjects carrying sufficient number of risky combinatory genotype sets in the pie have very high possibility of disease onset.

### Minimum and sufficient component causes

From Figure 5, the number of risky combinatory genotypes involved in a genetic causal pie which ranged from hundreds to thousands in the 14 gene clusters is rather high. By analyzing the functions of genes involved in a gene cluster, we found that many of them perform similar functions, and thus some of the genes may be redundant to the causal pie. An intuitive guess for such a redundancy is LD among SNPs (in different genes). We thus checked the LD between all SNPs in each of the 14 clusters using PLINK and retained only one of the gene pairs if their associated SNPs were found to have LD (D’ ≥ 0.9). After the LD reduction, the sizes of the 14 gene clusters were reduced in an average percentage of 96.82% without changing their classification accuracies.

To further remove the redundancy, we computed the minimum number of genes in each of the 14 clusters (via the genetic algorithm) without disrupting the classification accuracy of the LD-reduced gene set. We found substantial reduction in number of genes (23–42%, as detailed in Additional file 1: Table S6) for the 14 clusters, leaving the resultant number of genes in each cluster ranged from a few dozen to a few thousand. Furthermore, after the reduction, the cut-off percentage remained similar to that of the original gene set. However, some genes that seemed irrelevant or redundant in one dataset may have been crucial for HT identification in the other datasets. Therefore, whether such reductions sustain in larger datasets warrants further investigation.

We also used the gene expression data to compute the minimum genes in each cluster via the algorithm proposed in section 2.5 and Additional file 1: Method S3. We found the number of genes in each gene cluster can be tremendously reduced to around a couple of dozen while HT patients carrying risky combinatory genotypes can still be significantly discriminated (adjusted *p* < 10^-5^) from HT patients without carrying risky combinatory genotype, from NC subjects carrying risky combinatory genotypes and from NC subjects without carrying risky combinatory genotype (Additional file 1: Figure S6). Using the selected subsets of genes to repeat the previous genetic classification, we found the accuracies only decrease slightly (82.8%→78.9% for all datasets and 98.9→93.0% for all datasets but WTCCC_Affy500k, refer to Table 1), which implies that these gene may actually be important in each gene cluster. The selected gene symbols in the 14 gene clusters are provided in Additional file 4.

### Functional analysis of the gene clusters

Identifying key functions in the 14 gene clusters can help biologists to better understand the etiology of hypertension. The most intuitive approach is to look for the gene ontology (GO [36]) of the shared (major) genes (Additional file 1: Table S7). Of the 14 major genes, only PMS1, which is responsible for ATP and DNA bindings and is involved in repair of DNA mismatches, has been reported to associate with hypertension in African Americans [37]. Four major genes are associated with diseases frequently associated with hypertension, of which TMEM16A (also known as ANO1) is with pulmonary hypertension in rat [38], NINJ2 is with stroke [39], AKAP12 is with chronic kidney disease in Japanese individuals [40], and LTBP1 is with abdominal aortic aneurysm [41] and coronary atherosclerotic plaques [42]. Five major genes are linked to hypertension-related functions, of which CGREF1 and JPH1 are involved in calcium ion binding/transport, LPHN3 is in G-protein-coupled receptor signaling pathways, TNIK is in Wnt receptor signaling pathway, nervous system development and response to stress, and GRID2 is in glutamate signaling pathway, neuroactive ligand-receptor interaction and long-term depression. The remaining five major genes seem less hypertension-related. DNAH9 is responsible for ATP and nucleotide bindings and microtubule motor activity. Ectopic expression of C8orf72 (also known as FAM110B) proteins impaired cell cycle progression in G1 phase [43]. PKIB en-codes a protein which is a member of the cAMP-dependent protein kinase inhibitor family. NRXN1 functions in the vertebrate nervous system as cell adhesion molecules and receptors.

We also analyzed the functions, processes, and pathways of the other genes involved in the 14 gene clusters. We compared the GO information of the 14 gene clusters with that of 14 randomly generated, equal-sized, ones. Additional file 1: Table S8 lists the mechanisms in the 14 clusters that were significantly more abundant (*p* < 0.05) than those in the random sets. The majority of the listed mechanisms are known to highly related to hypertension, such as magnesium ion binding, calcium ion transport, central nervous system development, metabolic process and sodium ion transport. In addition, we utilized the genes selected from the gene expression data to find influential mechanisms in each individual gene cluster. We list in Additional file 1: Table S9 the influential pathways in which multiple genes in a cluster were involved whereas in Additional file 1: Table S10 we present the abundant functions, processes, and pathways in the individual gene clusters. From these tables, we found that gene clusters anchored by the major genes CGREF1, PMS1 and TNIK all had multiple genes in several cardiomyopathy-related pathways. Among the involved genes, a hypertension-candidate gene, CACNA1C, interacted with all three major genes suggesting its important role in cardiomyopathy-related hypertension. Moreover, multiple genes in gene clusters anchored by C8orf72, PMS1 and NRXN1 were found to involve in the metabolic pathways implying its influential role in these clusters. In addition, gene clusters anchored by TMEM16A, LPHN3 and GRID2 involved multiple neurotransmitter receptor genes contributing to the neuroactive ligand-receptor interaction pathway suggesting its possible link with hypertension. Finally, pathways such as axon guidance and Alzheimer’s disease also frequently involved in several gene clusters and their relationships with hypertension warrant further investigations.

Another attempt to identify influential disease mechanisms was to compare biomedical profiles among patient groups identified by the 14 gene clusters in Taiwan_Afft500k. We found that patients identified by the PKIB gene cluster had lower blood sodium levels (*p* = 0.038) than other patients, which coincided with situations in which the cluster consisted of more sodium channel activity genes than the others. In addition, the patients identified by the JPH1 gene cluster had lower blood potassium levels (*p* = 0.026) than other patients in the dataset, and this cluster happened to include more outward rectifier potassium channel activity genes than the others. Moreover, the LPHN3 gene cluster that identified six patients whose urine potassium levels were higher than other patients in the dataset (*p* = 0.026) contained abundant genes with clustering of voltage-gated potassium channels, potassium channel regulator activity, and calcium-activated potassium channel activity.

### Existence of alternative component causes in a genetic causal pie

We demonstrated that the 14 gene clusters resulting from the training datasets were reproducible in test datasets of different platforms through gene match (see Figures 3 and 4). Although the different sets of 14 clusters obtained from different platforms involved the same genes and mostly formed distinct gene-subject clusters, there was no direct proof that these gene clusters were the same across platforms. For example, in the DNAH9 gene cluster, we did not know whether the SNP pairs obtained from different platforms could identify the same group of HT patients.

To address this issue, we tested the 14 gene clusters on 46 HT patients for whom there existed both Affymetrix500k and Affymet-rix100k data and on 200 HT patients who had both Affymetrix500k and Illumina550k data (not used in the test datasets). As shown in the upper panel of Additional file 1: Figure S7, the HT patients identified in the Affymetrix500k data differed from those identified in the Affymetrix100k data. Similarly, in the lower panel, the HT patients identified in the Affymetrix500k data differed from those identified in the Illumina550k data. Both results indicated that, even with the same gene pairs, a gene cluster that consisted of different SNP pairs (used by different platforms) identified different groups of patients. The above results seemed to suggest that a genetic causal pie that involves multiple genes can involve different genetic variants (i.e., SNPs). If all the influential SNPs were genotyped, however, then the percentage of HT patients identified by the gene clusters could be increased.

## Discussion

We have developed a gene cluster construction algorithm for complex diseases, starting from finding influential gene pairs followed by grouping them into gene clusters. Each of the constructed gene clusters consisted of multiple gene pairs that were featured by a major gene interacting with multiple contributing genes and identified a similar group of patients. On an application to young-onset hypertension, our algorithm successfully constructed multiple reproducible gene clusters; each identified a distinct group of subjects. Our result demonstrated that the identification capability of such a gene cluster was resulted from a combinational effect of the gene pairs rather than the major gene. Also, only small percentage (3.18%) of contributing genes in a gene cluster exhibited LD. As a result, these gene clusters were highly susceptible to link with certain disease mechanisms of hypertension. Furthermore, the algorithm exhibited robustness (> 85% of the top 15 gene clusters remained unchanged) to criterion change, sample size change, and disease prevalence change.

The constructed gene clusters resemble Rothman’s causal pie model in that each gene cluster can be regarded as a causal pie with each risky combinatory genotype set of a gene pair in the cluster representing a component cause in the pie (a slice of the pie). And for each subject, the probability of ascertaining a disease increase dramatically as sufficient number of risky combinatory genotypes is carried. Multiple gene clusters, each of which identified a distinct group of subjects, imply multiple causal pies (disease mechanisms) for young-onset hypertension and may help to identify disease sub-types. Such a multi-causal pie-multi-component model provides an explanation of why conventional GWAS approaches in which all hypertensives were considered as a single group in comparison with the normotensives usually resulted in few significant genetic markers with poor reproducibility.

In this work, we presented the 14 large gene clusters constructed by our algorithm. These gene clusters were reproducible not only in Taiwanese and Caucasian populations but also across multiple genotyping platforms. In addition, they identified 19.3% of HT patients in all the datasets and 41.8% if the WTCCC_Affy500k was excluded for lack of biomedical profiles. Although 11.3% (with or without WTCCC_Affy500k) of NC subjects also carried risky combinatory genotypes in the gene clusters, they carried less risky combinatory genotypes than HT patients. After applying a suitable threshold to the number of risky combinatory genotypes in each gene cluster, we can further discriminate the HT patients from the NC subjects with an accuracy of 82.8% (sensitivity = 0.68 and specificity = 0.93) for all datasets and with an accuracy of 98.9% (sensitivity = 0.98 and specificity = 1.0) if the WTCCC_Affy500k was excluded.

The number of genes involved in the 14 gene clusters ranged from a few hundred to a few thousand. The meaning of such large number of genes is not clear. However, since multiple genes with similar functions are often involved in a given cluster and the influence of SNP variation is usually small, it is likely that it takes accumulative effects of multiple genes of the same functions and those of multiple pathways to lead to development of hypertension. Canalization [44], which measures the ability of a population to produce the same phenotype regardless of variability in its environment or genotype, may provide an explanation for the large number of genetic causal pies as well as the large number of component causes in a pie. Indeed, canalization values are high in most biological systems, implying that evolutionary forces select for traits that promote canalization which would ensure a normal blood pressure. Therefore, minor/moderate genetic or environment perturbations may not substantively impact biological systems. They need to be accumulated in some particular fashion and amount so as to cause malfunction in a biological system.

We have also listed in Additional file 1: Table S7–S10 some important functions, processes and pathways that are related to the 14 gene clusters. According to our gene-gene interaction model, the mechanisms that are related to a shared gene (Additional file 1: Table S7) should have strong associations with those that abundantly appeared in the gene cluster (Additional file 1: Table S9). To name a few, in the LTBP1 gene cluster, calcium ion binding/transport regulated by LTBP1 may be related to metabolism of lipids and lipoproteins that is attributed by two other genes in the cluster; in the TMEM16A gene cluster, calcium and chloride ion binding regulated by TMEM16A may be associated with purine metabolism, neuroactive ligand-receptor interaction and signaling by GPCR; in the LPHN3 gene cluster, G-protein-coupled receptor activity regulated by LPHN3 may interact with axon guidance, diabetes pathways and neuroactive ligand-receptor interaction; in the TNIK gene cluster, the stress response regulated by TNIK may be linked to arrhythmogenic right ventricular cardiomyopathy, dilated cardiomyopathy, hypertrophic cardiomyopathy and vascular smooth muscle contraction. However, further gene mapping endeavors are needed to depict detailed mechanisms in these gene clusters.

Owing to the difficulty of incorporating environment-gene with gene-gene interactions, in the present study, we only focused on constructing genetic causal pies for young-onset hypertension. With the genetic causal pies identified, we can then combine environmental factors, for example using the algorithm proposed by Hoffmann [45] or by Liao [46], to further explore the interactions between genetic and environmental factors and thus to better depict the hypertension etiology.

## Conclusions

In the present work, we developed a three-stage algorithm to construct gene clusters resembling Rothman’s causal pies, starting from finding influential gene pairs followed by grouping them and trained/tested on four large/diverse international databases. The constructed clusters, featured by a major gene interacting with many other genes, reproduced in Taiwanese and Caucasian populations and across multiple genotyping platforms. A total of 19.3–41.8% hypertensives were identified by 14 largest clusters and 68.8–95.7% of them carried sufficient amount of risky genotypes in at least one cluster. Furthermore, not only 10 of the 14 major genes but also many other genes in the clusters perform hypertension-related functions. Our results provide insights into polygenic aspect of hypertension etiology.

## Availability of supporting data

Additional files 1, 2, 3, 5, 4 and MATLAB files that generate Figures 3, 4, 5 in this manuscript are available at http://ms.iis.sinica.edu.tw/genetic_causal_pies/index.htm. The C++ codes that can be run on Linux clusters for computing all possible disease SNP pairs between two datasets and some other related MATLAB codes are also provided in the URL.

## Abbreviations

GWAS: Genome-wide association study; SNP: Single-nucleotide polymorphism; YOH: Young-onset hypertension; FHS: Framingham heart study; WTCCC: Wellcome trust case control consortium; THCCG: Taiwan Han Chinese cell and genome bank; HT: Hypertensive; NC: Normotensive control; GO: Gene ontology; FIS: Frequently identified subjects; MFIS: Most frequently identified subjects; LD: Linkage disequilibrium.

## Competing interests

The authors declare that they have no competing interests.

## Authors’ contributions

KSL conceived the methodology of the gene clustering algorithm, developed related software for the computation and analysis, and wrote the most of the manuscript. HYY developed a software to extract related gene information from the NCBI gene database. CHL participated in the development and improvement of the software for gene clustering. WLH and WHP conceived of the study and participated in its design and coordination. All authors read and approved the final manuscript.

## Supplementary Material

Additional file 1 **Methods S1–S5.** Figures S1–S7. Tables S1–S10. Click here for file

Additional file 2 Sample IDs used in the 5 datasets. Click here for file

Additional file 3 Detailed SNP rs numbers and the associated gene symbols in the gene clusters (at the cluster selection stage). Click here for file

Additional file 4 The selected gene symbols in the 14 gene clusters. Click here for file

Additional file 5 Detailed SNP rs numbers and the associated gene symbols in each of the gene clusters (at the component pruning stage). Click here for file

## References

[B1] Altshuler D Daly MJ Lander ES Genetic mapping in human disease Science 2008 322 5903 881 888 10.1126/science.1156409 18988837 PMC2694957

[B2] McCarthy MI Abecasis GR Cardon LR Goldstein DB Little J Ioannidis JP Hirschhorn JN Genome-wide association studies for complex traits: consensus, uncertainty and challenges Nat Rev Genet 2008 9 5 356 369 10.1038/nrg2344 18398418

[B3] Frazer KA Murray SS Schork NJ Topol EJ Human genetic variation and its contribution to complex traits Nat Rev Genet 2009 10 4 241 251 19293820 10.1038/nrg2554

[B4] Hardy J Singleton A Genomewide association studies and human disease N Engl J Med 2009 360 17 1759 1768 10.1056/NEJMra0808700 19369657 PMC3422859

[B5] Moore JH Asselbergs FW Williams SM Bioinformatics challenges for genome-wide association studies Bioinformatics 2010 26 4 445 455 10.1093/bioinformatics/btp713 20053841 PMC2820680

[B6] Pan WH Lynn KS Chen CH Wu YL Lin CY Chang HY Using endophenotypes for pathway clusters to map complex disease genes Genet Epidemiol 2006 30 2 143 154 10.1002/gepi.20136 16437587

[B7] Kohara K Tabara Y Nakura J Imai Y Ohkubo T Hata A Soma M Nakayama T Umemura S Hirawa N Identification of hypertension-susceptibility genes and pathways by a systemic multiple candidate gene approach: the millennium genome project for hypertension Hypertens Res: official journal of the Japanese Society of Hypertension 2008 31 2 203 212 10.1291/hypres.31.203 18360038

[B8] Lynn KS Li LL Lin YJ Wang CH Sheng SH Lin JH Liao W Hsu WL Pan WH A neural network model for constructing endophenotypes of common complex diseases: an application to male young-onset hypertension microarray data Bioinformatics 2009 25 8 981 988 10.1093/bioinformatics/btp106 19237446 PMC2666815

[B9] Zerba KE Ferrell RE Sing CF Genotype-environment interaction: apolipoprotein E (ApoE) gene effects and age as an index of time and spatial context in the human Genetics 1996 143 1 463 478 8722796 10.1093/genetics/143.1.463 PMC1207278

[B10] Sing CF Stengard JH Kardia SL Genes, environment, and cardiovascular disease Arterioscler Thromb Vasc Biol 2003 23 7 1190 1196 10.1161/01.ATV.0000075081.51227.86 12730090

[B11] Musani SK Shriner D Liu N Feng R Coffey CS Yi N Tiwari HK Allison DB Detection of gene x gene interactions in genome-wide association studies of human population data Hum Hered 2007 63 2 67 84 10.1159/000099179 17283436

[B12] Cordell HJ Detecting gene-gene interactions that underlie human diseases Nat Rev Genet 2009 10 6 392 404 19434077 10.1038/nrg2579 PMC2872761

[B13] Moore JH Williams SM Epistasis and its implications for personal genetics Am J Hum Genet 2009 85 3 309 320 10.1016/j.ajhg.2009.08.006 19733727 PMC2771593

[B14] Rothman KJ Causes Am J Epidemiol 1976 104 6 587 592 998606 10.1093/oxfordjournals.aje.a112335

[B15] Rothman KJ Greenland S Causation and causal inference in epidemiology Am J Public Health 2005 95 Suppl 1 S144 S150 16030331 10.2105/AJPH.2004.059204

[B16] Mongeau JG Heredity and blood pressure in humans: an overview Pediatr Nephrol 1987 1 1 69 75 10.1007/BF00866887 3153263

[B17] Pan WH Chen JW Fann C Jou YS Wu SY Linkage analysis with candidate genes: the Taiwan young-onset hypertension genetic study Hum Genet 2000 107 3 210 215 10.1007/s004390000365 11071381

[B18] Dawber TR Meadors GF Moore FE Jr Epidemiological approaches to heart disease: the Framingham study Am J Public Health Nations Health 1951 41 3 279 281 10.2105/AJPH.41.3.279 14819398 PMC1525365

[B19] Wellcome Trust Case Control C Genome-wide association study of 14,000 cases of seven common diseases and 3,000 shared controls Nature 2007 447 7145 661 678 10.1038/nature05911 17554300 PMC2719288

[B20] Pan WH Fann CS Wu JY Hung YT Ho MS Tai TH Chen YJ Liao CJ Yang ML Cheng AT Han Chinese cell and genome bank in Taiwan: purpose, design and ethical considerations Hum Hered 2006 61 1 27 30 10.1159/000091834 16534213

[B21] Riva A Kohane IS SNPper: retrieval and analysis of human SNPs Bioinformatics 2002 18 12 1681 1685 10.1093/bioinformatics/18.12.1681 12490454

[B22] Neuman RJ Rice JP Two-locus models of disease Genet Epidemiol 1992 9 5 347 365 10.1002/gepi.1370090506 1427023

[B23] Marchini J Donnelly P Cardon LR Genome-wide strategies for detecting multiple loci that influence complex diseases Nat Genet 2005 37 4 413 417 10.1038/ng1537 15793588

[B24] Fisher RA Sir The correlation between relatives on the supposition of Mendelian inheritance T Roy Soc Edin 1918 52 399 433

[B25] Armitage P Berry G Matthews JNS Statistical methods in medical research 2001 4 Malden, MA: Blackwell Science

[B26] Kooperberg C Ruczinski I Identifying interacting SNPs using Monte Carlo logic regression Genet Epidemiol 2005 28 2 157 170 10.1002/gepi.20042 15532037

[B27] Ritchie MD Hahn LW Roodi N Bailey LR Dupont WD Parl FF Moore JH Multifactor-dimensionality reduction reveals high-order interactions among estrogen-metabolism genes in sporadic breast cancer Am J Hum Genet 2001 69 1 138 147 10.1086/321276 11404819 PMC1226028

[B28] Nelson MR Kardia SL Ferrell RE Sing CF A combinatorial partitioning method to identify multilocus genotypic partitions that predict quantitative trait variation Genome Res 2001 11 3 458 470 10.1101/gr.172901 11230170 PMC311041

[B29] Nunkesser R Bernholt T Schwender H Ickstadt K Wegener I Detecting high-order interactions of single nucleotide polymorphisms using genetic programming Bioinformatics 2007 23 24 3280 3288 10.1093/bioinformatics/btm522 18006552

[B30] Motsinger-Reif AA Dudek SM Hahn LW Ritchie MD Comparison of approaches for machine-learning optimization of neural networks for detecting gene-gene interactions in genetic epidemiology Genet Epidemiol 2008 32 4 325 340 10.1002/gepi.20307 18265411

[B31] Chen SH Sun J Dimitrov L Turner AR Adams TS Meyers DA Chang BL Zheng SL Gronberg H Xu J A support vector machine approach for detecting gene-gene interaction Genet Epidemiol 2008 32 2 152 167 10.1002/gepi.20272 17968988

[B32] Zhang Y Liu JS Bayesian inference of epistatic interactions in case–control studies Nat Genet 2007 39 9 1167 1173 10.1038/ng2110 17721534

[B33] Kang G Yue W Zhang J Cui Y Zuo Y Zhang D An entropy-based approach for testing genetic epistasis underlying complex diseases J Theor Biol 2008 250 2 362 374 10.1016/j.jtbi.2007.10.001 17996908

[B34] Purcell S Neale B Todd-Brown K Thomas L Ferreira MA Bender D Maller J Sklar P de Bakker PI Daly MJ PLINK: a tool set for whole-genome association and population-based linkage analyses Am J Hum Genet 2007 81 3 559 575 10.1086/519795 17701901 PMC1950838

[B35] Neale BM Sham PC The future of association studies: gene-based analysis and replication Am J Hum Genet 2004 75 3 353 362 10.1086/423901 15272419 PMC1182015

[B36] Barrell D Dimmer E Huntley RP Binns D O’Donovan C Apweiler R The GOA database in 2009--an integrated Gene Ontology Annotation resource Nucleic Acids Res 2009 37 Database issue D396 D403 18957448 10.1093/nar/gkn803 PMC2686469

[B37] Adeyemo A Gerry N Chen G Herbert A Doumatey A Huang H Zhou J Lashley K Chen Y Christman M A genome-wide association study of hypertension and blood pressure in African Americans PLoS Genet 2009 5 7 e1000564 10.1371/journal.pgen.1000564 19609347 PMC2702100

[B38] Forrest AS Joyce TC Huebner ML Ayon RJ Wiwchar M Joyce J Freitas N Davis AJ Ye LD Duan DYD Increased TMEM16A-encoded calcium-activated chloride channel activity is associated with pulmonary hypertension Am J Physiol-Cell Ph 2012 303 12 C1229 C1243 10.1152/ajpcell.00044.2012 PMC3532492 23034390

[B39] Ikram MA Seshadri S Bis JC Fornage M DeStefano AL Aulchenko YS Debette S Lumley T Folsom AR van den Herik EG Genomewide association studies of stroke N Engl J Med 2009 360 17 1718 1728 10.1056/NEJMoa0900094 19369658 PMC2768348

[B40] Yoshida T Kato K Yokoi K Oguri M Watanabe S Metoki N Yoshida H Satoh K Aoyagi Y Nozawa Y Association of gene polymorphisms with chronic kidney disease in Japanese individuals Int J Mol Med 2009 24 4 539 547 19724895 10.3892/ijmm_00000263

[B41] Thompson AR Cooper JA Jones GT Drenos F van Bockxmeer FM Biros E Walker PJ van Rij AM Golledge J Norman PE Assessment of the association between genetic polymorphisms in transforming growth factor beta, and its binding protein (LTBP), and the presence, and expansion, of Abdominal Aortic Aneurysm Atherosclerosis 2010 209 2 367 373 10.1016/j.atherosclerosis.2009.09.073 19897194

[B42] Oklu R Hesketh R Wicky S Localization of latent transforming growth factor-beta binding protein-1 (LTBP1) in human coronary atherosclerotic plaques Arterioscl Throm Vas 2010 30 11 E253 E253 10.1253/circj.cj-10-0334 21071877

[B43] Hauge H Patzke S Aasheim HC Characterization of the FAM110 gene family Genomics 2007 90 1 14 27 10.1016/j.ygeno.2007.03.002 17499476

[B44] Waddington CH Canalization of development and genetic assimilation of acquired characters Nature 1959 183 4676 1654 1655 10.1038/1831654a0 13666847

[B45] Hoffmann K Heidemann C Weikert C Schulze MB Boeing H Estimating the proportion of disease due to classes of sufficient causes Am J Epidemiol 2006 163 1 76 83 16293718 10.1093/aje/kwj011

[B46] Liao SF Lee WC Weighing the causal pies in case–control studies Ann Epidemiol 2010 20 7 568 573 10.1016/j.annepidem.2010.04.003 20538201

