# Supplementary material for: Construction of gene clusters resembling genetic causal mechanisms for common complex disease with an application to young-onset hypertension
Source: BMC Genomics. 2013 Jul 23;14:497. doi: 10.1186/1471-2164-14-497 (PMC3751083; doi:10.1186/1471-2164-14-497)
Supplement: Additional file 4 — The selected gene symbols in the 14 gene clusters. [file 1471-2164-14-497-S4.pdf]

Supplementary Materials for:

Construction of Gene Clusters Resembling Genetic Causal Mechanisms for Common Complex Disease with an Application to Young-Onset Hypertension

Ke-Shiuan Lynn[1], Chen-Hua Lu[1], Han-Ying Yang[2], Wen-Lian Hsu[1] and Wen-Harn Pan[3,4*]

[1] Institute of Information Science, Academia Sinica, Taipei, Taiwan

[2] Department of Genome Sciences, University of Washington, Seattle, U.S.A.

[3] Institute of Biomedical Sciences, Academia Sinica, Taipei, Taiwan

[4] National Health Research Institutes, Mialoli, Taiwan

Overview of the Supplementary Materials:

Supplementary Methods

1. Inclusion criteria HT patients and for NC subjects
2. Validation algorithm of the 14 gene clusters using gene expression data
3. Cluster visualization
4. Robustness evaluation of the gene cluster construction algorithm
5. Probability of a false positive gene cluster

Supplementary Figures

1. Detailed aspects of subjects identified by the 14 gene clusters in the Taiwanese test datasets
2. Demonstration of a gene cluster construction process
3. Percentage of overlaps in the top-*n* gene clusters (*n* = 5, 10, 15, 20, 30, 50, 100) with respect to changes in the proportion of the case population used in the analysis
4. Percentage of overlaps in the top-*n* gene clusters (*n* = 5, 10, 15, 20, 30, 50, 100) with respect to changes in sample size in FHS_Affy500k
5. Box plots of the combined gene expression values for four subject groups in the fourteen gene clusters.

Supplementary Tables

1. Allele frequencies of the SNP *rs16854417* (*SLC9A9*) in different datasets
2. Numbers of overlapping genes between the 14 gene clusters
3. Percentage of HT patients who carried risky combinatory genotypes in each gene cluster among all patients in each dataset and percentage of NC subjects who carried risky combinatory genotypes in each gene cluster among all NC subjects in each dataset
4. Number of classification errors in the two training datasets evaluated at the validation sets of a five-fold validation procedure
5. Number of component causes in each gene cluster after LD reduction and after redundancy removal
6. Selected gene ontology of the 14 major genes
7. Mechanisms that were observed more/less frequently (P < 0.05) in the 14 gene clusters than in the 14 randomly generated, equal-sized, gene sets
8. Influential pathways in the individual gene cluster
9. Abundant functions, processes, and pathways in the individual gene clusters

Supplementary Methods

1. Inclusion criteria HT patients and for NC subjects

- HT patients: Subjects who satisfied all the listed criteria were included.
  - FHS
    1. Hypertension diagnosis: (i) Subject systolic blood pressure (SBP) ≥ 140 mmHg or diastolic blood pressure (DBP) ≥ 90 mmHg in at least two of four measurements (no subject was selected in the gen3 cohort under this criterion because only one BP measurement was available). (ii) Subject diagnosed as an HT patient at any examination.
    2. Body mass index (BMI) ≤ 35, blood sugar < 126 (not checked for gen3 cohort), no hard congenital heart disease (hard CHD), and not a diabetes patient.
    3. Age at onset, 20–50 years.
  - Taiwan YOH study
    1. Hypertension diagnosis: (i) Subject SBP ≥ 140 mmHg or DBP ≥ 90 mmHg in at least two consecutive visits in 2 months. (ii) Subject taking at least one anti-hypertensive medication. (iii) Not a secondary HT patient.
    2. BMI ≤ 35, blood sugar < 126, and hemoglobin A1c (HbAIC) < 7 (not an obese or diabetes patient).
    3. Age 20–50 years.
- NC subjects: Subjects who satisfied all the listed criteria were included.
  - FHS
    1. BP: Subject with a normal mean BP and has no more than one measure of SBP/DBP exceeding 120/80 mmHg.
    2. BMI ≤ 35.
    3. Blood sugar < 126 (not checked for gen3 cohort in FHS), and no hard CHD.
  - THCCG
    1. BP: Same criteria as used for FHS.
    2. BMI ≤ 35.
    3. HbAIC < 7 (not a diabetes patient).

2. Probability of a false positive gene cluster

We attempt to calculate the probability of a false positive gene cluster that contains *k* non-LD SNP pairs and identifies *m* subjects in a population of *n* subjects. Assumining that the *k* non-LD SNP pairs according identify m_1, m_2, m_3, …, m_k subjects with all m_i, i = 1, 2, 3, …, *k* subjects being subsets of the *m* subjects,

For a SNP pair that identifies m_1 subjects in a population of *n*, there are $C(n, m_1)$ possible combinations where $C(n, m_1) = n!/(m_1!(n-m_1)!)$ and therefore the probability for the first SNP pair in the gene cluster to identify the m_1 subjects by chance is $1/C(n, m_1) \le m_1/n \le m/n$. For the gene cluster of *k* non-LD SNP pairs to be formed by chance, the probability is $1/(C(n, m_1)*C(n, m_2)* C(n, m_3)*…* C(n, m_k)) \le (m_1* m_2*m_3* …*m_k)/n^k \le (m/n)^k$. In conclusion, the probability of a false positive gene cluster that contains *k* non-LD SNP pairs and identifies *m* subjects in a population of *n* subjects bounded above by $(m/n)^k$.

3. Validation algorithm of the 14 gene clusters using gene expression data

Let *hyp_mtx* be the hypertensive part and *nor_mtx* be the normotensive part of the data in which rows represent different genes and columns represent different subjects.

For each gene cluster

Let PID_{hyp_risky} be the indices of HT patients in *hyp_mtx* who carried risky combinatory genotypes and $PID_{hyp\_norisky}$ be those of HT patients who did NOT carry risky combinatory genotypes.

Let PID_{nor_risky} be the indices of NC subjects in *nor_mtx* who carried risky combinatory genotypes and $PID_{nor\_norisky}$ be those of NC subjects who did NOT carry risky combinatory genotypes.

Let GID_{shared} be the index of shared gene.

Set *hyp_vec* = *hyp_mtx*(GID_{shared},:).

Set *nor_vec* = *nor_mtx*(GID_{shared},:).

p1 = t-test(*hyp_vec*(PID_{hyp_risky}), *hyp_vec*($PID_{hyp\_norisky}$));

p2 = t-test(*hyp_vec*(PID_{hyp_risky}), *nor_vec*(PID_{nor_risky}));

p3 = t-test(*hyp_vec*(PID_{hyp_risky}), *nor_vec*($PID_{nor\_norisky}$));

$p_t = p1 + p2 + p3$;

Set *minp* =*tmpp*= p_t;

While *minp* ≧ *tmpp*

For each gene $i \neq GID_{shared}$ in the gene list

hyp_tmp = *hyp_vec*+*hyp_mtx*(*i*,:);

nor_tmp = *nor_vec*+*nor_mtx*(*i*,:);

$pp1_i$ = t-test(*hyp_tmp*(PID_{hyp_risky}), *hyp_tmp*($PID_{hyp\_norisky}$));

$pp2_i$ = t-test(*hyp_tmp*(PID_{hyp_risky}), *nor_tmp*(PID_{nor_risky}));

$pp3_i$ = t-test(*hyp_tmp*(PID_{hyp_risky}), *nor_tmp*($PID_{nor\_norisky}$));

hyp_tmp = *hyp_vec*-*hyp_mtx*(*i*,:);

nor_tmp = *nor_vec*-*nor_mtx*(*i*,:);

$np1_i$ = t-test(*hyp_tmp*(PID_{hyp_risky}), *hyp_tmp*($PID_{hyp\_norisky}$));

$np2_i$ = t-test(*hyp_tmp*(PID_{hyp_risky}), *nor_tmp*(PID_{nor_risky}));

$np3_i$ = t-test(*hyp_tmp*(PID_{hyp_risky}), *nor_tmp*($PID_{nor\_norisky}$));

$p_i = \min(pp1_i + pp2_i + pp3_i, np1_i + np2_i + np3_i)$;

If $p_i < tmpp$

tmpp= p_i;

best_id=i;

End If

End For

If *tmpp* < *minp*

Minp = *tmpp;*

hyp_vec = *hyp_vec*+*hyp_mtx*(*best_id*,:);

nor_vec = *nor_vec*+*nor_mtx*(*best_id*,:);

End If

End While

End For

4. Cluster visualization

We developed the following steps to generate gene-subject cluster plots for the demonstration of Rothman's genetic causal pies:

Step 1 Construct a binary matrix for each dataset in which each row in the matrix represents a SNP pair and a non-zero element indicates a subject carrying a risky combinatory genotype associated with the SNP pair.

Step 2 Reorder rows in the matrix such that SNP pairs with a shared gene are grouped together.

Step 3 Sort the resulting gene clusters by their size in descending order.

Step 4 Merge rows (SNP pairs) of the same gene pairs in a gene cluster into a single row using the "OR" operator if a similar group of subjects is identified.

Step 5 Starting from the largest gene clusters, group columns that represent subjects carrying risky combinatory genotypes in the gene cluster.

5. Robustness evaluation of the gene cluster construction algorithm

We tested the robustness of our gene cluster construction algorithm to small changes in criteria of the risky combinatory genotype. Because the clusters were selected at the first stage, we changed the criteria used at this stage. Apart from the original setting of 2.0% of the case population, we first tested the algorithm after increasing the setting to 2.5% and then decreasing it to 1.8% and 1.5%. These proportions were selected so as to change the numbers of cases in the two training datasets. That is, compared with the original 2% of the case population (7 and 4 cases in FHS_Affy500k and Taiwan_Affy500k, respectively), the 2.5%, 1.8% and 1.5% of case population corresponded to (8 and 5), (6 and 4) and (5 and 3) cases in the two datasets. To compute the similarity of the two lists of gene clusters, we first ranked the gene clusters in descending order based on the cluster size and then compared the shared genes corresponding to the top n gene clusters (n = 5, 10, 15, 20, 30, 50, 100). The top n gene clusters of the two lists were said to have 100% overlap if their corresponding shared genes were the same (regardless of the ranking order).

We also tested the robustness of the developed gene cluster construction algorithm to changes in sample size. Because the Taiwan_Affy500k was already small in terms of NC sample size (184 NC subjects), and further reducing it could introduce a considerable amount of false-positive SNP pairs, we only reduced the sample size of FHS_Affy500k to 90%, 70%, and 50% of its original size in our experiments. For each size, three sub-datasets were constructed, each of which was randomly drawn from the original dataset. The similarity between the gene clusters computed from the reduced sub-dataset and those computed from the original dataset was evaluated via the same procedure as that used to evaluate the effect of criterion changes.

Supplementary Figures

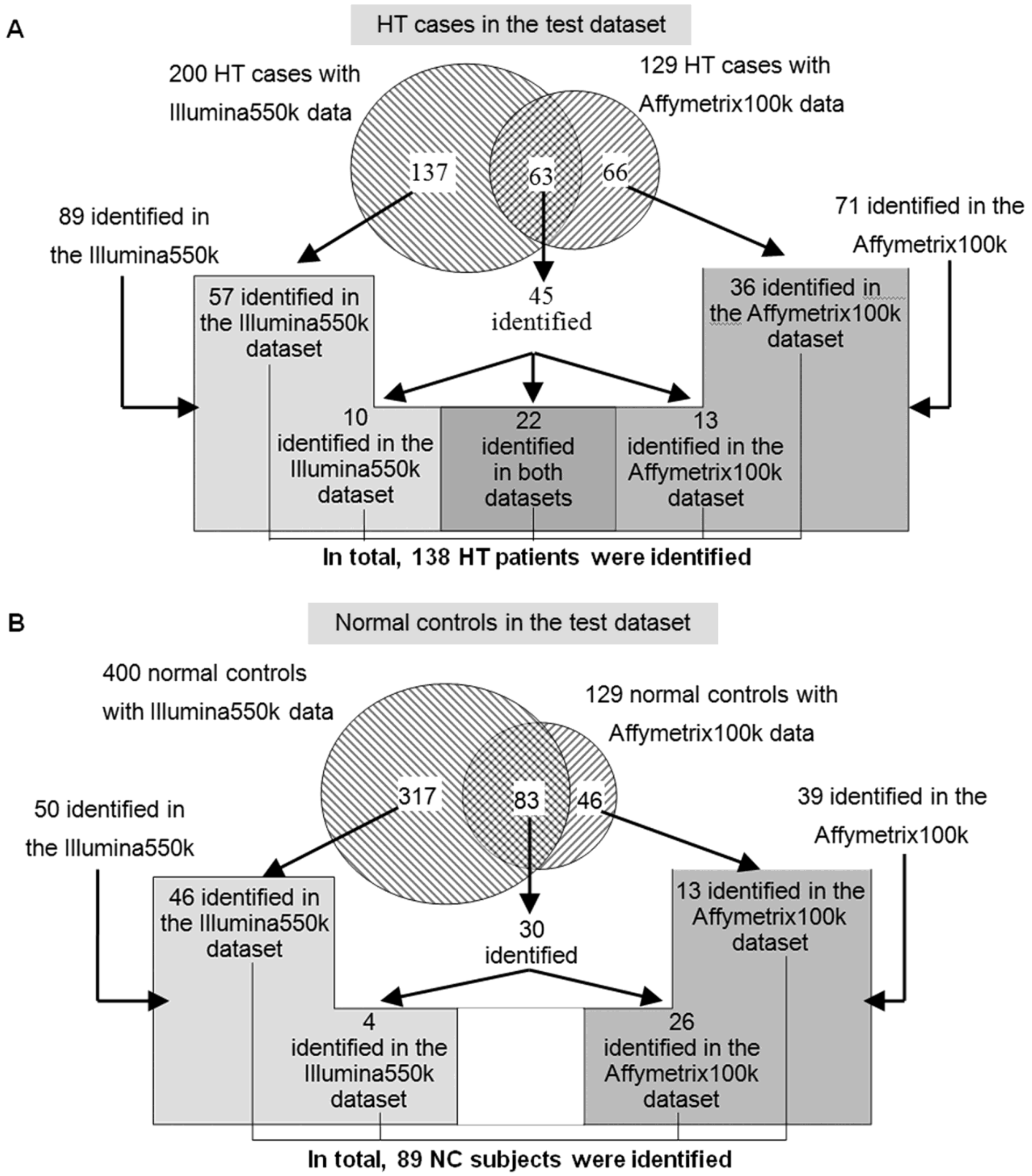

Supplementary Fig. 1. Detailed aspects of subjects identified by the 14 gene clusters in the Taiwanese test datasets; The Taiwanese test datasets include Taiwan_Affy100k and Taiwan_Illu550k. The upper panel (A) is for HT cases and the lower panel (B) is for NC subjects.

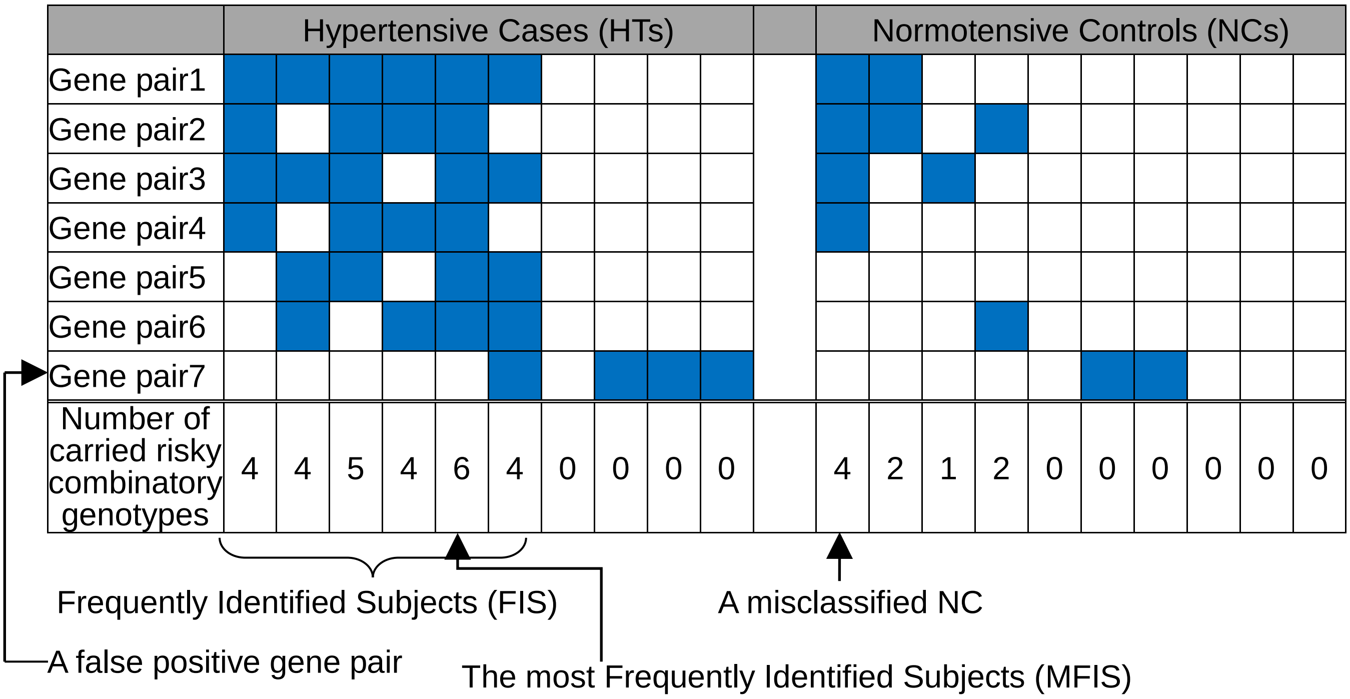

Supplementary Fig. 2. Demonstration of a gene cluster construction process

The top-6 hypertensive patients are frequently identified subjects (FISs) whereas the fifth one is the most frequently identified subjects (MFIS). On the other hand, there are 7 gene pairs in the above gene cluster. Of the 7 gene pairs, the gene pair 1, which identifies 6 patients, is the most effective, whereas the gene pair 7 identifies a different group of patients and thus will be removed from the gene cluster. According to the number of risky combinatory genotypes carried by the FISs, the sufficient number of component causes (risky combinatory genotypes) is 4. Under such a threshold, the first normotensive control is misclassified.

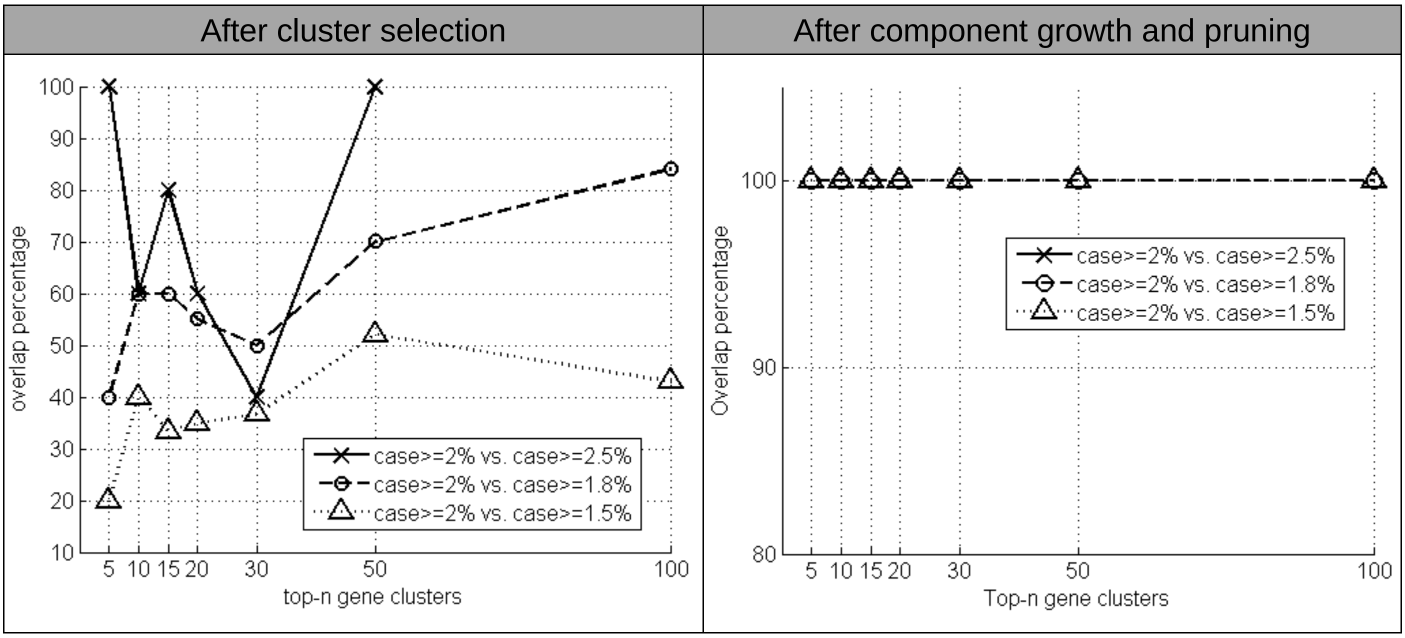

Supplementary Fig. 3. Percentage of overlaps in the top-*n* gene clusters (*n* = 5, 10, 15, 20, 30, 50, 100) with respect to changes in the proportion of the case population used in the analysis

The left panel shows results after the cluster selection stage of our cluster construction algorithm whereas the right panel shows results after component growth and pruning stages. It should be noted that the solid data line was cut off at *n* = 50 because the criterion became too stringent and only 51 gene clusters were qualified.

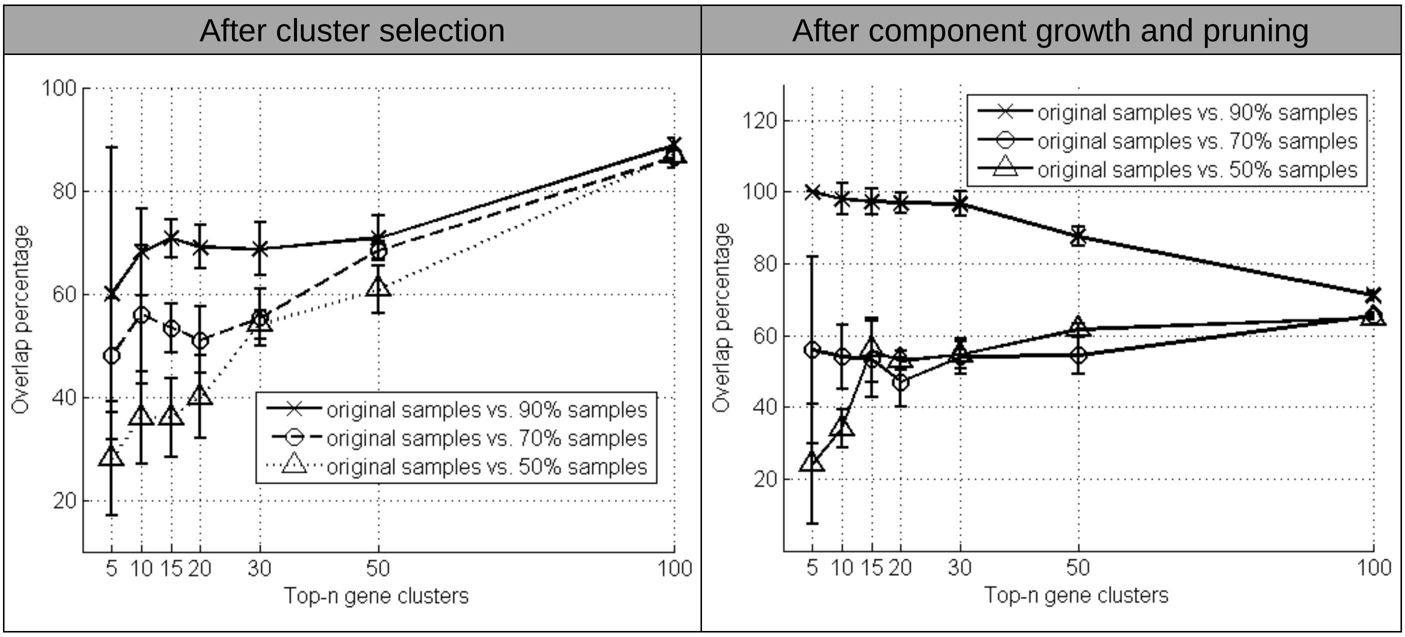

Supplementary Fig. 4. Percentage of overlaps in the top-*n* gene clusters (*n* = 5, 10, 15, 20, 30, 50, 100) with respect to changes in sample size in FHS_Affy500k

The left panel shows results after the cluster selection stage of our cluster construction algorithm whereas the right panel shows results after component growth and pruning stages.

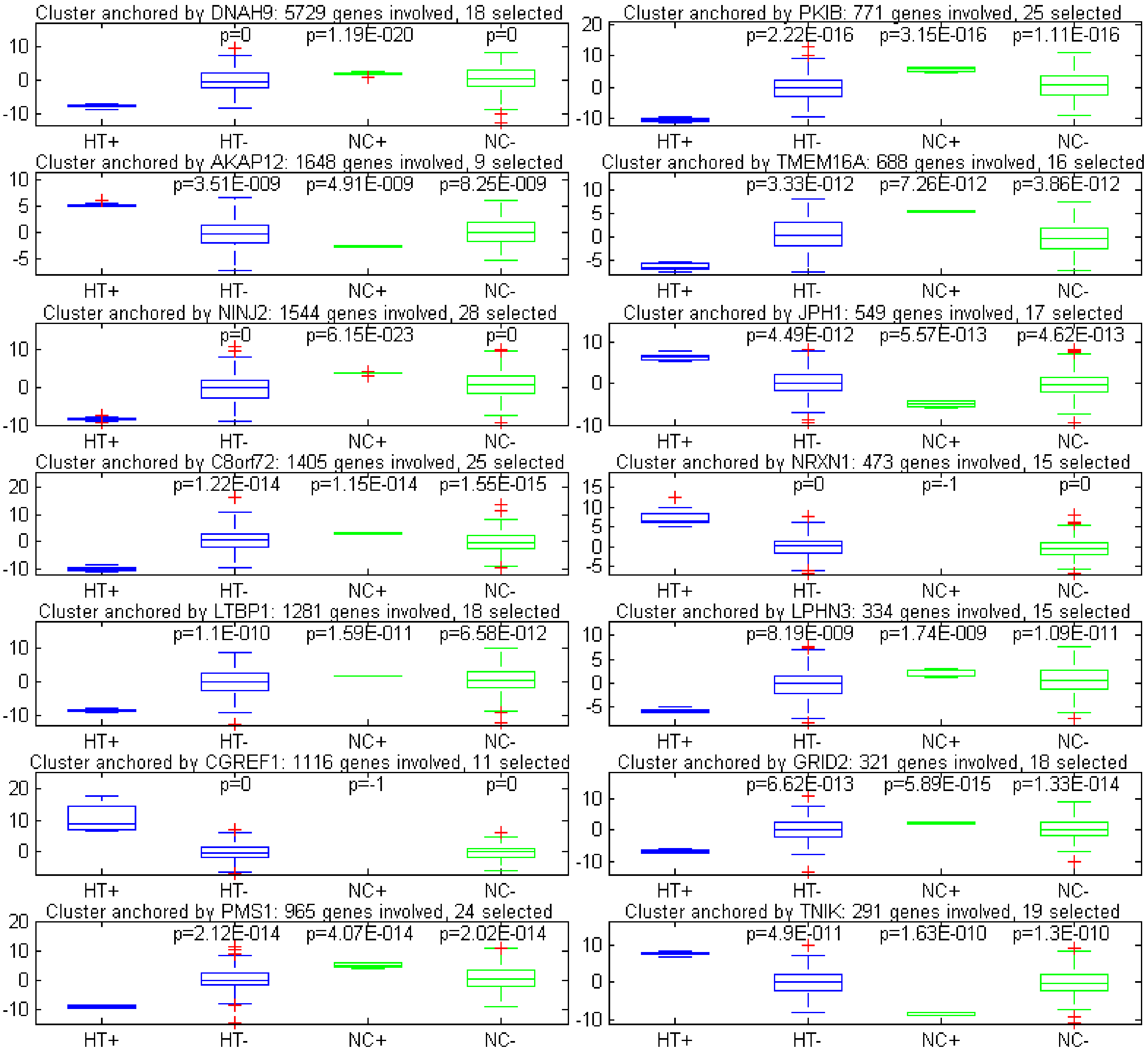

Supplementary Fig. 5. Box plots of the combined gene expression values for four subject groups in the fourteen gene clusters. Of the four subject groups, HT+ represents hypertensives carrying risky combinatory genotypes, HT- represents hypertensives without carrying risky combinatory genotypes, NC+ denotes normotensives carrying risky combinatory genotypes and NC- denotes normotensives without carrying risky combinatory genotypes. The *p* value indicates the t-test result of the corresponding subject group with respective to the HT+ group (*p* = -1 indicates no subject in the corresponding group).

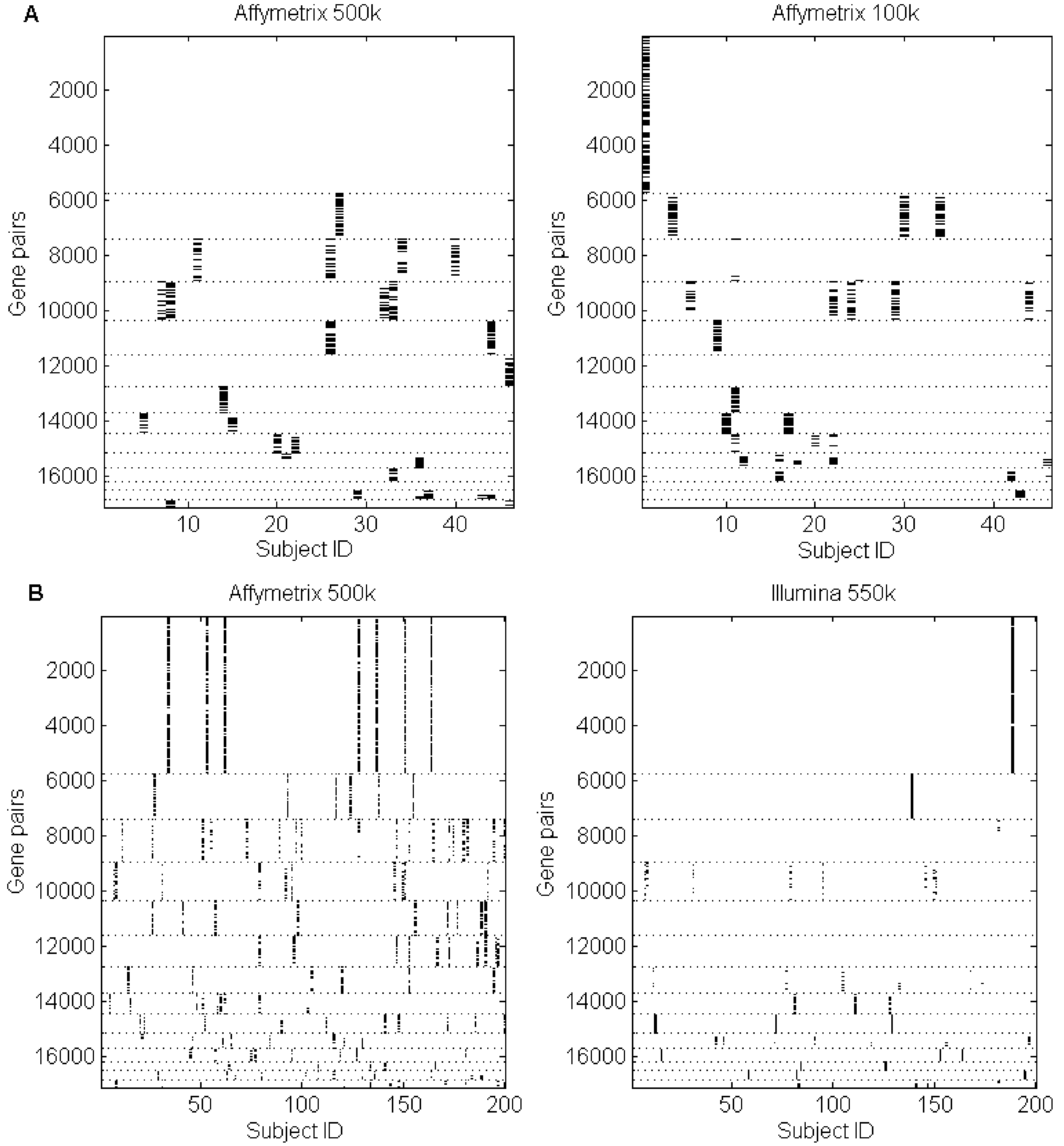

Supplementary Fig 6 Gene clusters consisting of the same gene pairs but different SNP pairs may identify different groups of patients

Overlapped patients in different genotyping platforms were used to test whether the same gene clusters detected from different platforms identify the same group of patients (i.e., whether the different SNP pairs selected from different platforms have LD): (A) 46 HT overlapped patients in both Affymetrix 500k and Affymetrix 100k data were tested; (B) 200 HT overlapped patients in both Affymetrix 500k and Illumina550k data were tested.

Supplementary Tables

Supplementary Table 1. Allele frequencies of the SNP *rs16854417* (*SLC9A9*) in different datasets

Ethnicity		Caucasian			Asian	
Datasets		HapMap-CEU	FHS_Affy500k	WTCCC_Affy500k	HapMap-HCB	Taiwan_Affy500k
Sample size		120	Case:305, Control:2881	Case:2001, Control:3004	90	Case:200, Control:184
Allele frequency	**CC**	**0%**	**total: 0.09% (case:0.98%, control:0%)**	**total: 0.04% (case:0.05%, control:0.03%)**	**0%**	**total: 1.3% (case:2.0%, control:0.54%)**
	CG	3.3%	total: 3.7% (case:3.28%, control:3.75%)	total: 2.29% (case:1.95%, control:2.73%)	22.2%	total: 11.7% (case:11.5%, control:11.96%)
	GG	96.7%	total: 94.4% (case:93.1%, control:94.1%)	total: 97.20% (case:97.35%, control:97.07%)	77.8%	total: 86.2% (case:85.0%, control:87.5%)

CEU: U.S. Utah residents with ancestry from northern and western Europe, HCB: Han Chinese in Beijing, China

Supplementary Table 2. Numbers of overlapping genes between the 14 gene clusters

	DNAH9	*AKAP12*	*NINJ2*	*C8orf72*	*LTBP1*	*CGREF1*	*PMS1*	*PKIB*	*TMEM16A*	*JPH1*	*NRXN1*	*LPHN3*	*GRID2*	*TNIK*
DNAH9	5729	403	222	258	260	184	183	128	125	84	88	72	64	44
AKAP12	403	1648	60	47	69	44	41	17	28	10	14	16	14	10
NINJ2	222	60	1544	63	58	24	48	37	27	36	10	6	15	8
C8orf72	258	47	63	1405	58	38	45	29	24	16	11	4	21	10
LTBP1	260	69	58	58	1281	31	28	26	17	16	15	8	8	7
CGREF1	184	44	24	38	31	1116	31	30	17	8	9	9	7	8
PMS1	183	41	48	45	28	31	965	15	23	16	2	4	6	4
PKIB	128	17	37	29	26	30	15	771	7	9	8	2	5	3
TMEM16A	125	28	27	24	17	17	23	7	688	11	8	3	4	10
JPH1	84	10	36	16	16	8	16	9	11	549	1	3	1	3
NRXN1	88	14	10	11	15	9	2	8	8	1	473	12	3	0
LPHN3	72	16	6	4	8	9	4	2	3	3	12	334	0	0
GRID2	64	14	15	21	8	7	6	5	4	1	3	0	321	2
TNIK	44	10	8	10	7	8	4	3	10	3	0	0	2	291

Supplementary Table 3. Percentage of HT patients who carried risky genotypes in each gene cluster among all patients in each dataset and percentage of NC subjects who carried risky genotypes in each gene cluster among all NC subjects in each dataset

No. of genes	Shared gene	**HT carrying risky genotypes (%)**					**NC carrying risky genotypes (%)**				
		Training		Test		Total	Training		Test		Total
		Cau	TW	Cau	TW	% (No.)	Cau	TW	Cau	TW	% (No.)
**1**	*SLC9A9*	0.98	2.00	0.05	N/A	0.32 (8)	0	0.54	0.03	N/A	0.03 (2)
**2–55**	Omitted due to insufficient gene pairs for evaluation of frequently identified subjects										
**291**	*TNIK*	1.97	4.50	0.55	4.14	1.33 (37)	0.76	4.35	0.83	0.45	0.87 (57)
**321**	*GRID2*	1.31	6.00	0.30	5.26	1.30 (36)	0.38	4.89	0.53	1.12	0.63 (41)
**334**	*LPHN3*	3.28	3.00	1.75	3.76	2.20 (61)	0.80	0.54	1.13	1.79	1.01 (66)
**473**	*NRXN1*	2.62	4.00	1.00	5.64	1.84 (51)	0.56	1.63	1.07	0.45	0.81 (53)
**549**	*JPH1*	2.62	4.00	0.15	7.52	1.41 (39)	0.62	5.43	0.40	1.79	0.74 (48)
**688**	*TMEM16A*	2.62	5.50	1.50	5.26	2.27 (63)	0.62	2.17	1.53	3.14	1.26 (82)
**771**	*PKIB*	2.30	4.50	0.45	4.51	1.33 (37)	0.45	7.07	0.80	0.90	0.83 (54)
**965**	*PMS1*	3.28	3.00	1.30	6.02	2.09 (58)	0.62	4.89	1.63	0.90	1.23 (80)
**1,116**	*CGREF1*	2.62	5.00	0.05	2.26	0.90 (25)	0.83	0.54	0.07	0.90	0.48 (31)
**1,281**	*LTBP1*	2.30	4.50	0.55	3.76	1.33 (37)	0.59	3.26	0.93	1.12	0.86 (56)
**1,405**	*C8orf72*	2.62	5.00	1.15	6.02	2.06 (57)	0.69	5.43	0.80	2.47	1.00 (65)
**1,544**	*NINJ2*	2.62	9.00	0.50	7.52	2.02 (56)	0.59	5.98	0.37	2.47	0.77 (50)
**1,648**	*AKAP12*	2.62	3.00	0.65	4.51	1.41 (39)	0.66	1.09	0.67	1.35	0.72 (47)
**5,729**	*DNAH9*	3.28	3.50	1.35	9.02	2.45 (68)	0.49	1.09	0.97	3.81	0.95 (62)

Cau: Caucasian, TW: Taiwanese

Supplementary Table 4 Number of classification errors in the two training datasets evaluated at the validation sets of a five-fold validation procedure

	FHS_Affy500k					Taiwan_Affy500k				
	Validation set1	Validation set2	Validation set3	Validation set4	Validation set5	Validation set1	Validation set2	Validation set3	Validation set4	Validation set5
DNAH9	0	0	0	0	1	0	0	0	0	1
AKAP12	1	0	0	0	0	0	1	0	0	0
NINJ2	0	0	0	0	1	0	0	0	0	1
C8orf72	0	1	0	0	0	0	2	1	0	2
LTBP1	0	0	0	0	1	0	0	1	0	0
CGREF1	1	0	0	0	0	1	0	0	0	0
PMS1	0	0	0	1	0	0	0	0	1	0
PKIB	0	0	1	0	0	1	0	0	0	0
TMEM16A	0	1	0	0	0	0	1	0	1	0
JPH1	0	0	1	0	0	0	1	0	0	0
NRXN1	0	0	0	1	0	0	0	0	0	1
LPHN3	0	0	0	1	1	0	0	0	2	0
GRID2	0	0	0	0	0	0	1	0	0	0
TNIK	0	1	0	0	0	1	0	0	0	0
Overall Classification accuracy	99.91%					98.96%				

Supplementary Table 5 Number of component causes in each gene cluster after LD reduction and after redundancy removal

	Number of component causes		
	After cluster construction	After LD reduction	After redundancy removal
DNAH9	5729	5521 (96.21%)	2427 (42.36%)
AKAP12	1648	1585 (96.18%)	624 (37.86%)
NINJ2	1544	1497 (96.96%)	578 (37.44%)
C8orf72	1405	1371 (97.58%)	524 (37.30%)
LTBP1	1281	1236 (96.49%)	453 (35.36%)
CGREF1	1116	1080 (96.77%)	410 (36.74%)
PMS1	965	943 (97.72%)	347 (35.96%)
PKIB	771	754 (97.80%)	265 (34.37%)
TMEM16A	688	666 (96.80%)	207 (30.09%)
JPH1	549	530 (96.54%)	170 (30.97%)
NRXN1	473	458 (96.83%)	114 (24.10%)
LPHN3	334	316 (94.61%)	82 (24.55%)
GRID2	321	310 (96.57%)	82 (25.55%)
TNIK	291	286 (98.28%)	68 (23.37%)

Supplementary Table 6. Selected gene ontology of the 14 major genes

Shared gene	Location	No. of genes	Selected gene ontology
DNAH9	17p12	5729	**Function**: ATP binding, microtubule motor activity
AKAP12	6q24-q25	1648	**Process**: G-protein-coupled receptor protein signaling pathway
NINJ2	12p13	1544	**Process**: nervous system development, tissue regeneration<br>**Phenotype**: genome-wide association studies of stroke
C8orf72	8q12.1	1405	
LTBP1	2p22-p21	1281	**Function**: calcium ion binding, growth factor binding<br>**Pathway**: TGF-beta signaling pathway
CGREF1	2p23.3	1116	**Function**: calcium ion binding<br>**Process**: response to stress
PMS1	2q31-q33; 2q31.1	965	**Function**: ATP binding<br>**Process**: DNA mismatch repair
PKIB	6q22.31	771	**Function**: cAMP-dependent protein kinase inhibitor activity<br>**Process**: negative regulation of protein kinase activity
TMEM16A	Chromosome 5	688	**Function**: calcium ion binding, chloride ion binding
JPH1	8q21	549	**Function**: structural constituent of muscle<br>**Process**: calcium ion transport into cytosol, regulation of ryanodine-sensitive calcium-release channel activity
NRXN1	2p16.3	473	**Function**: metal ion binding<br>**Process**: axon guidance<br>**Pathway**: cell adhesion molecules (CAMs)<br>**Phenotype**: susceptibility to autism
LPHN3	4q13.1	334	**Function**: G-protein-coupled receptor activity, sugar binding<br>**Process**: G-protein-coupled receptor protein signaling pathway
GRID2	4q22	321	**Function**: extracellular-glutamate-gated ion channel activity<br>**Process**: glutamate signaling pathway<br>**Pathway**: neuroactive ligand-receptor interaction, long-term depression
TNIK	3q26.2-q26.31	291	**Function**: protein serine/threonine kinase activity, small GTPase regulator activity<br>**Process**: Wnt receptor signaling pathway, activation of JNKK activity, nervous system development, response to stress

Supplementary Table 7. Mechanisms that were observed more/less frequently ($p < 0.05$) in the 14 gene clusters than in the 14 randomly generated, equal-sized, gene sets

	Functions, processes and pathways	Gene ratios in the 14 gene clusters	Gene ratios in the 14 random sets	*p*-value
Functions	Acyltransferase activity	0.0049±0.0019	0.0074±0.0017	1.05×10^{-3}
	**Calmodulin binding**	**0.0136±0.0037**	**0.0055±0.0049**	$\mathbf{3.40\times10^{-5}}$
	Kinase activity	0.0039±0.0041	0.0072±0.0026	1.77×10^{-2}
	Magnesium ion binding	0.0061±0.0059	0.0099±0.0032	4.13×10^{-2}
	Motor activity	0.0052±0.0016	0.0020±0.0030	1.31×10^{-3}
	Olfactory receptor activity	0.0056±0.0082	0.0152±0.0120	2.04×10^{-2}
	Receptor binding	0.0127±0.0020	0.0073±0.0083	2.68×10^{-2}
Processes	**Activation of protein kinase C activity by G-protein-coupled receptor protein signaling pathway**	**0.0040±0.0020**	**0.0001±0.0020**	$\mathbf{1.90\times10^{-5}}$
	Axonogenesis	0.0062±0.0026	0.0016±0.0030	1.92×10^{-4}
	Calcium ion transport	0.0130±0.0034	0.0056±0.0057	3.05×10^{-4}
	**Central nervous system development**	**0.0129±0.0033**	**0.0037±0.0050**	$\mathbf{5.00\times10^{-6}}$
	**Ion transport**	**0.0477±0.0080**	**0.0281±0.0066**	$\mathbf{1.67\times10^{-7}}$
	**Metabolic process**	**0.0217±0.0048**	**0.0071±0.0072**	$\mathbf{1.00\times10^{-6}}$
	Mitosis	0.0028±0.0050	0.0105±0.0045	2.20×10^{-4}
	Protein homo-oligomerization	0.0049±0.0012	0.0022±0.0036	1.31×10^{-2}
	Response to drug	0.0080±0.0097	0.0149±0.0042	2.25×10^{-2}
	Sensory perception of smell	0.0081±0.0101	0.0196±0.0037	4.55×10^{-4}
	Sensory perception of sound	0.0085±0.0072	0.0014±0.0045	4.16×10^{-3}
	Sodium ion transport	0.0088±0.0041	0.0052±0.0049	4.24×10^{-2}
Pathways	Adherens junction	0.0070±0.0030	0.0020±0.0059	8.67×10^{-3}
	Cell adhesion molecules (CAMs)	0.0131±0.0047	0.0057±0.0059	1.10×10^{-3}
	Dilated cardiomyopathy	0.0083±0.0076	0.0027±0.0041	2.22×10^{-2}
	Huntington's disease	0.0046±0.0059	0.0088±0.0022	2.05×10^{-2}
	Hypertrophic cardiomyopathy (HCM)	0.0080±0.0076	0.0029±0.0040	3.64×10^{-2}
	Natural killer cell mediated cytotoxicity	0.0027±0.0064	0.0069±0.0031	3.72×10^{-2}
	Phosphatidylinositol signaling system	0.0066±0.0056	0.0023±0.0051	4.24×10^{-2}
	Signaling in Immune system	0.0099±0.0034	0.0132±0.0038	2.14×10^{-2}
	Signaling by NGF	0.0144±0.0038	0.0093±0.0051	6.00×10^{-3}
	Tight junction	0.0113±0.0053	0.0057±0.0068	2.32×10^{-2}

Note: In the above list, we only present mechanisms that are sufficient abundant (at least 6 of the 14 gene clusters had to contain ≥ 0.5% of all genes associated with a particular mechanism) in the 14 gene clusters.

Supplementary Table 8. Influential pathways in the individual gene cluster

Major gene	No. of Genes	Identified Patients	Influential Pathways
DNAH9	18	15	Axon guidance(2), Hemostasis(2)
AKAP12	9	8	
NINJ2	28	16	Axon guidance(2), ErbB signaling pathway(2), Focal adhesion(2), Regulation of actin cytoskeleton(3)
C8orf72	25	10	Alzheimer's disease(2), Calcium signaling pathway(2), Metabolic pathways(2)
LTBP1	18	9	Metabolism of lipids and lipoproteins(2)
CGREF1	11	4	Arrhythmogenic right ventricular cardiomyopathy(2), Axon guidance(2), Calcium signaling pathway(2), Cardiac muscle contraction(2), Dilated cardiomyopathy(2), Glutamatergic synapse(2), Hemostasis(2), Hypertrophic cardiomyopathy(2)
PMS1	24	11	Arrhythmogenic right ventricular cardiomyopathy(2), Axon guidance(2), Dilated cardiomyopathy(2), Hypertrophic cardiomyopathy(2), Metabolic pathways(3)
PKIB	25	11	Axon guidance(4)
TMEM16A	16	13	Neuroactive ligand-receptor interaction(2), Purine metabolism(3), Signaling by GPCR(2)
JPH1	17	13	
NRXN1	15	10	Axon guidance(2), Metabolic pathways(2)
LPHN3	15	9	Axon guidance(3), Diabetes pathways(2), Neuroactive ligand-receptor interaction(3)
GRID2	18	14	Axon guidance(2), Neuroactive ligand-receptor interaction(2), Synaptic Transmission(2)
TNIK	19	8	Alzheimer's disease(2), Arrhythmogenic right ventricular cardiomyopathy(2), Axon guidance(3), Dilated cardiomyopathy(2), Hypertrophic cardiomyopathy(2), Vascular smooth muscle contraction(2)

Note: Numbers in the parenthesis indicate the number of genes involved in the corresponding pathway.

Supplementary Table 9 Abundant functions, processes, and pathways in the individual gene clusters

Shared gene	No. of genes	Abundant mechanisms
DNAH9	5729	**Process**: positive regulation of insulin secretion++, positive regulation of stress-activated MAPK cascade++<br>**Pathway**: ubiquinone and other terpenoid-quinone biosynthesis++
AKAP12	1648	**Function**: glucuronosyltransferase activity++, copper ion binding++, sodium ion binding++<br>**Process**: positive regulation of cholesterol storage++

		**Pathway**: starch and sucrose metabolism+
NINJ2	1544	**Function**: G-protein-coupled receptor activity*, long-chain fatty acid-CoA ligase activity++, folic acid binding++, nucleoside: sodium symporter activity++, bile acid:sodium symporter activity++, lipid transporter activity++, very long-chain fatty acid-CoA ligase activity++<br>**Process**: homocysteine metabolic process++, energy reserve metabolic process++, folic acid and derivative metabolic process++, G-protein signaling, coupled to cGMP nucleotide second messenger++<br>**Pathway**: PPAR signaling pathway++, glyoxylate and dicarboxylate metabolism++, muscle contraction++
C8orf72	1405	**Function**: G-protein-coupled photoreceptor activity++<br>**Process**: T cell receptor signaling pathway++, positive regulation of T cell differentiation++, negative regulation of G-protein-coupled receptor protein signaling pathway++, regulation of T cell receptor signaling pathway++<br>**Pathway**: metabolism of lipids and lipoproteins*
LTBP1	1281	**Function**: SH3 domain binding**<br>**Process**: G-protein-coupled receptor protein signaling pathway*, positive regulation of inflammatory response++, glycogen metabolic process++, gluconeogenesis++, positive regulation of systemic arterial blood pressure++, folic acid and derivative biosynthetic process++, G-protein signaling, coupled to cGMP nucleotide second messenger++, glycerol transport++, glycerol metabolic process, response to fatty acid++<br>**Pathway**: signaling by VEGF++
CGREF1	1116	**Function**: magnesium ion binding*, voltage-gated sodium channel activity++<br>**Process**: muscle contraction*, Wnt receptor signaling pathway++, response to glucocorticoid stimulus++, positive regulation of fatty acid oxidation++, positive regulation of potassium ion transport++<br>**Pathway**: galactose metabolism++
PMS1	965	**Function**: insulin binding++, G-protein-coupled receptor binding++, sodium:dicarboxylate symporter activity++, insulin-like growth factor I binding++, insulin-like growth factor receptor binding++<br>**Process**: regulation of G-protein-coupled receptor protein signaling pathway++, insulin receptor signaling pathway++, negative regulation of insulin receptor signaling pathway++, blood vessel maturation++, negative regulation of glucose import++, insulin-like growth factor receptor signaling pathway++<br>**Pathway**: diabetes pathways**, metabolic pathways*, signaling by Wnt++
PKIB	891	**Function**: sugar binding*, calcium channel regulator activity++, triglyceride lipase activity++<br>**Process**: regulation of fatty acid oxidation++, detection of calcium ion++, negative regulation of inflammatory response++, JAK-STAT cascade++, low-density lipoprotein particle remodeling++, positive regulation of lipid storage++<br>**Pathway**: fructose and mannose metabolism++
TMEM16A	771	**Process**: regulation of muscle contraction++, cholesterol esterification++, negative regulation of smooth muscle cell proliferation++, negative regulation of blood coagulation++, lipopolysaccharide biosynthetic process++<br>**Pathway**: signaling by insulin receptor++, lipid digestion, mobilization, and transport++

JPH1	688	**Function**: calcium ion binding*, extracellular-glycine-gated chloride channel activity++, calcium: sodium antiporter activity++<br>**Process**: inflammatory response**, release of sequestered calcium ion into cytosol++, response to glucose stimulus++, patterning of blood vessels++, regulation of calcium ion transport++<br>**Pathway**: calcium signaling pathway*, glycerophospholipid metabolism++
NRXN1	549	**Function**: voltage-gated calcium channel activity**, sodium:phosphate symporter activity++, calcium-release channel activity++<br>**Process**: ventricular cardiac muscle cell differentiation++, regulation of cardiac muscle contraction by regulation of the release of sequestered calcium ion++<br>**Pathway**: arrhythmogenic right ventricular cardiomyopathy (ARVC)*, cardiac muscle contraction*, signaling by Rho GTPases*
LPHN3	473	**Function**: chloride channel activity**, voltage-gated chloride channel activity++, voltage-gated chloride channel activity++<br>**Process**: heart development**, carbohydrate metabolic process**, lipid metabolic process*, glucose metabolic process++, circadian rhythm++, response to calcium ion++, blood circulation++<br>**Pathway**: Wnt signaling pathway**, fatty acid metabolism++
GRID2	334	**Function**: phospholipid binding**, lipid binding*<br>**Process**: lipid catabolic process**, nervous system development*, potassium ion transport*, negative regulation of calcium ion transport via voltage-gated calcium channel activity++, cholesterol homeostasis++<br>**Pathway**: vascular smooth muscle contraction**, T cell receptor signaling pathway**, Jak-STAT signaling pathway**
TNIK	291	**Function**: voltage-gated calcium channel activity**, lipid binding**, voltage-gated ion channel activity++, cholesterol monooxygenase (side-chain-cleaving) activity++, inward rectifier potassium channel activity++, large conductance calcium-activated potassium channel activity++<br>**Process**: negative regulation of lipid catabolic process++, negative regulation of Wnt receptor signaling pathway++<br>**Pathway**: dilated cardiomyopathy**, hypertrophic cardiomyopathy (HCM)**, arrhythmogenic right ventricular cardiomyopathy (ARVC)*, cardiac muscle contraction*, T cell receptor signaling pathway*, MAPK signaling pathway++

Note: Three symbols indicate different levels of influence: “**” indicates that the ratio of associated genes is *at least 0.01* in the cluster and is *much higher (≥ mean + 2 SD)* than in the other gene clusters; “*” is similar to “**” except that the ratio is only *slightly higher (≥ mean + 1.5 SD)* than in the other gene clusters; “++” denotes that the ratio of associated genes is *between 0.002 and 0.01* in the cluster and is *much higher (≥ mean + 2 SD)* than in the other gene clusters.
